# Genome-wide mining seed-specific candidate genes from peanut for promoter cloning

**DOI:** 10.1371/journal.pone.0214025

**Published:** 2019-03-28

**Authors:** Cuiling Yuan, Quanxi Sun, Yingzhen Kong

**Affiliations:** 1 Key Laboratory for Tobacco Gene Resources, Tobacco Research Institute of Chinese Academy of Agricultural Sciences, Qingdao, Shandong, China; 2 Shandong Peanut Research Institute, Qingdao, Shandong, China; 3 Biotechnology Research Institute, Chinese Academy of Agricultural Sciences, Beijing, China; Institute of Genetics and Developmental Biology Chinese Academy of Sciences, CHINA

## Abstract

Peanut seeds are ideal bioreactors for the production of foreign recombinant proteins and/or nutrient metabolites. Seed-Specific Promoters (SSPs) are important molecular tools for bioreactor research. However, few SSPs have been characterized in peanut seeds. The mining of Seed-Specific Candidate Genes (SSCGs) is a prerequisite for promoter cloning. Here, we described an approach for the genome-wide mining of SSCGs via comparative gene expression between seed and nonseed tissues. Three hundred thirty-seven SSCGs were ultimately identified, and the top 108 SSCGs were characterized. Gene Ontology (GO) analysis revealed that some SSCGs were involved in seed development, allergens, seed storage and fatty acid metabolism. RY REPEAT and GCN4 motifs, which are commonly found in SSPs, were dispersed throughout most of the promoters of SSCGs. Expression pattern analysis revealed that all 108 SSCGs were expressed specifically or preferentially in the seed. These results indicated that the promoters of the 108 SSCGs may perform functions in a seed-specific and/or seed-preferential manner. Moreover, a novel SSP was cloned and characterized from a paralogous gene of SSCG29 from cultivated peanut. Together with the previously characterized SSP of the SSCG5 paralogous gene in cultivated peanut, these results implied that the method for SSCG identification in this study was feasible and accurate. The SSCGs identified in this work could be widely applied to SSP cloning by other researchers. Additionally, this study identified a low-cost, high-throughput approach for exploring tissue-specific genes in other crop species.

## Introduction

Peanut (*Arachis hypogaea* L., which is also referred to as groundnut) is one of the most important oil crop species worldwide and plays important roles in human nutrition [1]. Peanut seeds, which are rich in oleic acid, linoleic acid, proteins and other nutrients, are ideal bioreactors for the production of foreign recombinant proteins or other beneficial metabolites.

As important molecular tools, promoters are usually used in gene functional analysis [2–4] and are also widely used for plant quality improvement [5–8]. Seed-specific promoters (SSPs), which can drive the expression of foreign genes specifically in seeds, are of great importance for genetic engineering of seeds. SSPs have been widely applied in plant molecular pharming, such as that involving golden rice [8], purple endosperm rice [9], purple embryo maize [7] and fish oil canola [10]. The use of SSPs can avoid constitutive expression, which can harm plants [11–13]. Moreover, repetitive use of the same promoter when expressing multiple foreign proteins simultaneously is considered inadvisable owing to the likelihood of transcriptional silencing [14–16]. Therefore, additional peanut SSPs are needed to overexpress or knock down specific genes, regulate seed development, and modify seed content, especially to produce foreign recombinant proteins or secondary metabolites.

To date, few SSPs from peanut are available, and those that are available were identified from known genes expressed specifically in the seed [17–19]. Tissue-specific gene expression provides fundamental information for SSP mining. Several methods have been developed to analyze gene expression differences, such as subtractive hybridization [20], suppression subtractive hybridization [21], differential display reverse transcription PCR [22], and cDNA microarrays [23,24]. However, these methods are limited by their specific shortcomings; for example, only known genes can be recognized by microarray chips [23]. With the decreasing cost of transcriptome sequencing, comparative transcriptome sequencing has been widely used to analyze differences in gene expression [25–28]. The diploid peanut ancestors *Arachis duranensis* (AA) and *Arachis ipaensis* (BB) are considered the donors of the A and B subgenomes of the allotetraploid cultivated peanut *Arachis hypogaea* [1]. The release of *A*. *duranensis* and *A*. *ipaensis* genome sequences [1] made it convenient to obtain genetic information from cultivated peanut. Comparative transcriptome sequencing combined with peanut genome information is a powerful means of genome-wide mining of SSCGs for promoter cloning.

In this study, we described a genome-wide comparative transcriptome sequencing-based approach to identify SSCGs for SSP cloning in peanut. A total of 337 SSCGs were identified from peanut, and the top 108 SSCGs according to their Fragments Per Kilobase of transcript per Million mapped reads (FPKMs) were characterized. On the basis of semiquantitative RT-PCR analysis, 94 SSCGs were expressed in a seed-specific manner, and 14 SSCGs were expressed in a seed-preferential manner. One novel SSP was cloned and characterized to verify its seed specificity in transgenic *Arabidopsis*. Our results could be widely used in the identification of future peanut SSPs.

## Materials and methods

### Plant materials and RNA extraction

Plants of the cultivated peanut ‘Shitouqi’ were grown at the Laixi experimental station of the Shandong Peanut Research Institute during the summer of 2016. Leaves, roots, stems, pegs and pod shells were collected at the pod-maturing stage. Developing seeds were collected between 20 and 80 days after flowering. All tissues were flash frozen in liquid nitrogen and then stored at -80°C for transcriptome sequencing.

Total RNA was isolated from different tissues using TRIzol (Life Technologies, Carlsbad, CA, USA) reagent. The quality and quantity of each RNA sample were assayed using a NanoDrop device (Thermo Fisher, MA, USA).

### Illumina sequencing and in silico analysis

The RNA extracted from seeds at different development stages was mixed together as Sample I (seed), while the RNA from the leaves, roots, stems, pegs and pod shells were pooled in equimolar amounts as Sample II (nonseed). Both samples were treated and sequenced using an Illumina HiSeq^TM^ 2500 instrument at Gene Denovo Biotechnology Company (Guangzhou, China). Transcript reads containing adaptor sequences were cleaned, and low-quality reads were filtered and removed. The transcript reads of each sample were then mapped to the *A*. *duranensis* and *A*. *ipaensis* reference genomes [1] by TopHat2 [29].

The gene expression levels were normalized using FPKM methods. To mining SSCGs, the FPKM value of each transcript in Sample I was divided by the value in Sample II using Excel software. The FPKM values of the SSCGs that were less than 10 in Sample I or greater than 10 in Sample II as well as yield values greater than 50 were considered SSCGs. The SSCGs were subsequently listed according to their FPKM value.

### GO annotation, chromosomal location and cis-acting element analysis

Functional annotation and Gene Ontology (GO) analyses of the SSCGs were carried out using BLAST2GO (http://www.geneontology.org/). All SSCG sequences and chromosomal location information were obtained from the PeanutBase database (www.peanutbase.org). These genes were mapped onto the chromosome using the MapInspect software program (http://mapinspect.software.informer.com). To identify cis-acting elements, the 2500 bp promoter regions upstream of the ATG initiation codon of the SSCGs were identified using the New PLACE server (https://sogo.dna.affrc.go.jp/cgi_bin/sogo.cgi?lang=en&pj=640&action=page&page=newplace) [30].

### Phylogenetic analysis

To study the phylogenetic relationship of the selected SSCGs, multiple alignments of their DNA sequence were performed using the computer program ClustalW. Unrooted phylogenetic trees were constructed in accordance with the neighbor-joining (NJ) method using MEGA 6.0 software, and the bootstrap test was carried out with 1000 iterations.

### Expression analysis of SSCGs in *A*. *duranensis* and *A*. *ipaensis*

The FPKM data of the 108 selected SSCGs within 20 distinct tissues were retrieved from the work of Clevenger et al. [31]. The FPKM normalized read count data of the SSCGs were log2-transformed and displayed in the form of heat maps via HemI [32].

### Semiquantitative RT-PCR analysis in cultivated peanut

To confirm the tissue expression specificity in cultivated peanut further, RNA extracted from the leaves, roots, stems, pegs, pod shells and seeds were collected at the pod-maturing stage. Three independent RNA preparations were used for semiquantitative RT-PCR. Twenty-six amplification cycles were used to evaluate and quantify the differences among transcript levels. RT-PCR was performed using the peanut *Actin* gene as an internal control [33]. PCR was performed using 2*Easy Taq PCR SuperMix (TransGen Biotech, Beijing, China). The PCR conditions were as follows: one initial denaturation step of 94°C for 3 min; 26 cycles of 94°C for 30 s, 58°C for 30 s and 72°C for 30 s; and one final extension step of 72°C for 10 min. Three independent RNA preparations were used for semiquantitative RT-PCR. The primers used for these experiments are listed in S3 Table.

### Isolation of an SSP

Peanut genomic DNA was isolated from young leaves of the ‘Shitouqi’ cultivar using a DNAquick Plant System Kit (Tiangen, Beijing, China). Using AHSSP29-specific primers (S3 Table), we performed PCR with PrimeSTAR GXL DNA Polymerase (Takara, Dalian, China). The PCR products were separated by electrophoresis through a 1.5% agarose gel and purified using a gel extraction kit (TransGen Biotech, Beijing, China). All purified PCR products were subcloned into a pEASY-blunt simple vector (TransGen Biotech, Beijing, China). The DNA sequences were sequenced by the Shanghai Sangon Biotechnology Company (Shanghai, China).

The promoter fragment AHSSP29 of SSCG29 was excised from the pEASY-blunt simple vector with the restriction enzymes *Hin*dIII and *Bam*HI (Thermo Fisher, MA, USA) and ligated into the corresponding restriction sites of the plant transformation vector pBI121 to produce an AHSSP29::β-glucuronidase (GUS) construct.

### Generation of transgenic *Arabidopsis* plants

The recombinant binary plasmid was transferred to *Agrobacterium tumefaciens* strain GV3101, and kanamycin-resistant colonies were selected on medium containing 50 μg ml^-1^ kanamycin. A selected colony was grown to stationary phase at 28°C, and the cells were concentrated by centrifugation and then resuspended in a dipping solution that comprised 5% sucrose, 0.03% Silwet-77, and 10 mM MgCl_2_ [34]. The seeds were harvested and subsequently stored at room temperature. For screening, the seeds were sterilized in 75% (v/v) ethanol for 3 min and then 2.6% NaClO for 10 min, followed by several washes with sterile water. The transformants were screened on one-half-strength Murashige and Skoog (MS) medium that contained 50 μg ml^-1^ kanamycin.

### Transgene detection in the transgenic progeny of *Arabidopsis* and GUS histochemical staining

Kanamycin-resistant transgenic *Arabidopsis* plants were identified using *GUS* gene-specific primers (S3 Table). The positive transgenic plants were then selfed, after which homozygous T_2_ progeny were obtained.

The GUS activity was measured as described previously [35]. The samples were incubated with GUS staining buffer (0.1% Triton X-100, 2 mM 5-bromo-4-chloro-3-indolyl-β-D-glucuronide (X-Gluc), and cyclohexyl ammonium salt in 100 mM sodium phosphate buffer, pH 7.0) at 37°C overnight and then decolorized with 70% ethanol.

## Results

### Genome-wide mining of SSCGs via comparative transcriptome sequencing

To mining SSCGs, two samples of the cultivated peanut ‘Shitouqi’ (Sample I for seed samples and Sample II for nonseed samples) were used for transcriptome sequencing via an Illumina HiSeq^TM^ 2500 system. Approximately 10 Gb of sequence data (approximately 76.79 million reads from Sample I and 78.93 million reads from Sample II, each 300 bp in length) were obtained; after filtering the adaptor sequences and low-quality reads, approximately 75.37 and 77.81 million reads were used for transcriptome assembly, respectively (S1 Table). All of the reconstructed genes were aligned to the reference genome of *A*. *duranensis* and *A*. *ipaensis* [1] and were subsequently annotated. A comparative transcript profile was established based on the FPKM values of the assembly transcripts. Three hundred thirty-seven SSCGs were ultimately identified and designated sequentially as SSCG1 to SSCG337 according to their FPKM value. The detailed information of these SSCGs, including their gene symbol, chromosomal location, FPKM value and putative function(s), is listed in Table 1 and S2 Table. GO annotation was performed using BLAST2GO, and the 337 SSCGs were categorized with particular GO annotations (S1 Fig, Table 2). Expectedly, these SSCGs were enriched in metabolic process (120) and catalytic activity (108) GO terms, which suggested the presence of vigorous metabolic activity in the seed, in which fatty acids such as oleic acid are converted into linoleic acid by fatty acid desaturase [36]. To identify promoters that are strongly or specifically expressed in the seed, the most abundant top 108 SSCGs were chosen for further analysis. With the decreasing cost of transcriptome sequencing and the release of the peanut ancestor genome, comparative transcriptome sequencing has become an efficient approach for mining tissue-specific genes from peanut and other less studied crop species.

**Table 1:** List of 108 SSCGs identified from *A*. *duranensis* and *A*. *ipaensis* by comparative transcriptome sequencing.

ID	Gene symbol	Chromosomal location	Nonseed FPKM	Seed FPKM	Seed FPKM/Nonseed FPKM	Putative function
SSCG1	Araip.D61U9	B06:22151849..22156660	9.6	40464.62	3817.41698	Nutrient reservoir protein, putative
SSCG2	Aradu.F9TAJ	A06:1278068..1286732	7.9	29576.44	3743.85316	Nutrient reservoir protein, putative
SSCG3	Aradu.YGS80	A06:1778238..1782613	6.13	23996.24	3914.55791	Nutrient reservoir protein, putative
SSCG4	Araip.T82B5	B09:145805360..145809988	5.48	17844.62	3256.31752	Vicilin 47 kDa protein
SSCG5	Araip.WQE9Q	B06:19983167..19987898	3.65	11809.74	3235.54521	Nutrient reservoir, putative
SSCG6	Aradu.2H0R0	A09:111189111..111193663	2.85	11232.05	3941.07018	Allergen gly M Bd 28 kDa protein
SSCG7	Aradu.YBK6Q	A06:1263038..1268055	1.84	7346.02	3992.40217	Nutrient reservoir, putative
SSCG8	Aradu.B98FL	A02:14075236..14078402	1.15	4528.01	3937.4	Nutrient reservoir protein, putative
SSCG9	Araip.5JB56	B06:21966364..21970937	0.88	3850.03	4375.03409	Nutrient reservoir protein, putative
SSCG10	Aradu.I3E1J	A01:95190268..95194982	0.94	3292.1	3502.23404	PREDICTED: desiccation-related protein PCC13-62-like [*Glycine max*]
SSCG11	Araip.CF8RS	B01:132019082..132024234	0.93	2692.8	2895.48387	PREDICTED: desiccation-related protein PCC13-62-like [*Glycine max*]
SSCG12	Araip.4G9JR	B07:124167306..124175151	4.16	2538.22	610.149038	PREDICTED: uncharacterized protein LOC100803807 isoform X4 [*Glycine max*]
SSCG13	Araip.16S9Q	B06:1939514..1945848	0.71	2002.03	2819.76056	Allergen gly M Bd 28 kDa protein
SSCG14	Araip.DH1Z0	B08:1478391..1487297	1.74	1730.87	994.752874	Seed linoleate 9S-lipoxygenase
SSCG15	Araip.UPW6L	B06:13539947..13544474	0.58	1639.78	2827.2069	Short-chain dehydrogenase-reductase B
SSCG16	Araip.XV8NA	B09:121459378..121466147	0.91	1567.95	1723.02198	Caleosin-related family protein
SSCG17	Araip.930A9	B07:103614634..103618108	0.37	1391.54	3760.91892	Plant EC metallothionein-like protein
SSCG18	Aradu.L7CNH	A07:57016256..57019539	0.75	1093.32	1457.76	Dehydrin family protein
SSCG19	Araip.GWR7V	B06:3643904..3647892	0.38	1236.21	3253.18421	Seed maturation protein
SSCG20	Aradu.P54FB	A06:4694298..4698399	1.25	1207.86	966.288	Short-chain dehydrogenase-reductase B
SSCG21	Aradu.CPR44	A08:16217242..16225065	1.32	1127.39	854.083333	PREDICTED: uncharacterized protein LOC100803807 isoform X4 [*Glycine max*]
SSCG22	Araip.TR541	B07:64847542..64850823	0.27	935.31	3464.11111	Dehydrin family protein
SSCG23	Araip.E99Y9	B08:1487051..1496731	2.19	885.49	404.333333	Seed linoleate 9S-lipoxygenase
SSCG24	Aradu.A02RY	A06:12429384..12433425	0.36	868.98	2413.83333	Seed maturation protein
SSCG25	Aradu.8NU6I	A03:108778760..108781946	7.04	778.99	110.651989	Unknown protein
SSCG26	Araip.GVB7U	B02:104940525..104947120	0.1	764.27	7642.7	Adenine nucleotide α-hydrolase-like superfamily protein
SSCG27	Aradu.TC8DF	A09:100677241..100681879	0.15	749.56	4997.06667	Caleosin-related family protein
SSCG28	Aradu.DWL7L	A05:84098086..84102786	0.46	706.68	1536.26087	Water-selective transport intrinsic membrane protein 1
SSCG29	Aradu.YC8MH	A05:9432005..9436963	0.36	705.76	1960.44444	Nutrient reservoir, putative
SSCG30	Araip.MGW36	B05:10060638..10064989	0.44	699.31	1589.34091	Allergen gly M Bd 28 kDa protein
SSCG31	Araip.SK1EN	B06:21959550..21964666	0.11	660.71	6006.45455	Nutrient reservoir, putative
SSCG32	Aradu.UQE92	A07:7869609..7873957	0.41	630.02	1536.63415	Unknown protein
SSCG33	Araip.XXN6R	B05:14183132..14188591	0.37	607.63	1642.24324	PREDICTED: vacuolar-processing enzyme-like [*Glycine max*]
SSCG34	Araip.LJX8Z	B05:144093715..144097932	0.2	595.64	2978.2	Water-selective transport intrinsic membrane protein 1
SSCG35	Aradu.F1JZ5	A05:16468072..16472629	1.5	584.6	389.733333	Hydroxysteroid dehydrogenase 5
SSCG36	Aradu.1QI16	A06:1256618..1260495	0.17	529.57	3115.11765	Nutrient reservoir, putative
SSCG37	Aradu.WX5KP	A08:23296802..23303303	2.52	517.02	205.166667	Seed linoleate 9S-lipoxygenase
SSCG38	Aradu.7S7IW	A03:1743950..1749685	0.23	495.13	2152.73913	Allergen gly M Bd 28 kDa protein
SSCG39	Araip.S2F61	B06:13664618..13669683	0.37	489.34	1322.54054	PREDICTED: basic 7S globulin [*Glycine max*]
SSCG40	Araip.YX1UI	B03:132064839..132069075	0.24	484.56	2019	Alkyl hydroperoxide reductase/Thiol-specific antioxidant/Mal allergen
SSCG41	Araip.LRG7E	B06:21098054..21102262	0.4	482.27	1205.675	Nutrient reservoir, putative
SSCG42	Aradu.G7AM5	A06:1289026..1294066	0.07	472.41	6748.71429	Nutrient reservoir, putative
SSCG43	Araip.213GN	B08:22780923..22784155	1.49	442.09	296.704698	Defensin related
SSCG44	Aradu.I953D	A06:14925068..14929464	0.09	413.62	4595.77778	Vicilin 47 kDa protein
SSCG45	Araip.IGC50	B06:22165994..22175182	0.17	399.54	2350.23529	Nutrient reservoir, putative
SSCG46	Araip.I9427	B03:3690475..3698992	0.42	399.48	951.142857	Allergen gly M Bd 28 kDa protein
SSCG47	Araip.C23HG	B03:106887073..106891066	0.05	396.23	7924.6	Short-chain dehydrogenase-reductase B
SSCG48	Araip.Z0Q6Q	B10:134692303..134702146	3.72	394.85	106.142473	Annexin 8
SSCG49	Araip.18621	B06:62925430..62926840	0.04	386.85	9671.25	35 kDa seed maturation protein [*Glycine max*]
SSCG50	Aradu.Y7IVD	A05:13381777..13387424	0.33	378.86	1148.06061	PREDICTED: vacuolar-processing enzyme-like [*Glycine max*]
SSCG51	Araip.K8QIE	B10:4310337..4313223	1.81	373.44	206.320442	Maturation protein pPM32 [*Glycine max*]
SSCG52	Araip.PTL0N	B06:11564210..11570467	2.1	364.85	173.738095	Nodulin MtN21/EamA-like transporter family protein
SSCG53	Araip.8Y88R	B07:15516942..15521234	2.43	363.4	149.547325	Sugar transporter SWEET
SSCG54	Araip.FWQ8E	B10:10536351..10540643	2.43	363.4	149.547325	Sugar transporter SWEET
SSCG55	Aradu.QK6K1	A02:91132444..91136523	0.27	343.33	1271.59259	Adenine nucleotide α-hydrolase-like superfamily protein
SSCG56	Araip.U0WFW	B09:145740761..145744766	5.31	343.23	64.6384181	Seed maturation protein
SSCG57	Aradu.QV0LR	A03:131080569..131083815	0.31	329.27	1062.16129	1-Cysteine peroxiredoxin 1
SSCG58	Aradu.7HS2D	A06:5723487..5729852	2.23	328.45	147.286996	Nodulin MtN21/EamA-like transporter family protein
SSCG59	Araip.534K5	B05:17042251..17046905	0.04	327.32	8183	Oxidoreductase, short-chain dehydrogenase/reductase family protein, expressed
SSCG60	Araip.YF7VP	B10:2845816..2849609	0.14	326.69	2333.5	Protein of unknown function
SSCG61	Araip.55BM4	B06:4524371..4531866	0.06	323.4	5390	PREDICTED: probable galactinol-sucrose galactosyltransferase 2-like isoform X2 [*Glycine max*]
SSCG62	Aradu.ZQ8HD	A06:57618672..57622857	0.3	320.3	1067.66667	35 kDa Seed maturation protein [*Glycine max*]
SSCG63	Aradu.F3KB2	A09:120096458..120099936	0.06	320.12	5335.33333	AWPM-19-like family protein
SSCG64	Araip.Z2VYZ	B05:4533706..4536889	0.07	307.96	4399.42857	PREDICTED: ethylene-responsive transcription factor 13-like [*Glycine max*]
SSCG65	Aradu.KPI4B	A06:110359168..110362750	4.95	301.2	60.8484848	Gibberellin-regulated family protein
SSCG66	Aradu.HX36X	A08:32778205..32783203	0.24	300.89	1253.70833	Seed biotin-containing protein SBP65 [*Glycine max*]
SSCG67	Aradu.TVV1L	A10:6209012..6213515	1.76	299.09	169.9375	Sugar transporter SWEET
SSCG68	Aradu.G1YNF	A09:114688277..114693267	1.22	293.18	240.311475	Fatty acid desaturase 2
SSCG69	Aradu.Y6LUX	A09:1576235..1582427	1.13	288.63	255.424779	Late embryogenesis abundant protein (LEA) family protein
SSCG70	Aradu.N27YB	A09:70208795..70214429	5.56	287.19	51.6528777	PREDICTED: uncharacterized protein LOC100802932 isoform X2 [Glycine *max*]
SSCG71	Aradu.BXD3B	A03:104961788..104965971	0.25	285.35	1141.4	Oxidoreductase, short-chain dehydrogenase/reductase family protein
SSCG72	Aradu.KQ35F	A02:91130378..91134093	0	285.2	-	-
SSCG73	Aradu.Z8JSI	A03:127984231..127991012	0.04	268.05	6701.25	Flowering locus protein T
SSCG74	Aradu.9S6MI	A06:4648907..4652669	0.62	267.51	431.467742	PREDICTED: basic 7S globulin [*Glycine max*]
SSCG75	Aradu.XDS84	A06:1477479..1481775	0.13	263.9	2030	Nutrient reservoir, putative
SSCG76	Araip.27I5U	B03:125840273..125843558	0.39	261.23	669.820513	Gibberellin-regulated protein n = 1 Tax = *Medicago truncatula*
SSCG77	Araip.FYJ9U	B01:123329168..123333431	4.19	259.66	61.9713604	Late embryogenesis abundant protein (LEA), putative/LEA protein, putative
SSCG78	Araip.XR8KB	B10:129753362..129759594	4.64	252.97	54.5193966	PREDICTED: probable 2-Oxoglutarate/Fe(II)-dependent dioxygenase-like [*Glycine max*]
SSCG79	Aradu.9Z0RX	A02:93238735..93243417	0.18	249.63	1386.83333	Late embryogenesis abundant protein (LEA), putative
SSCG80	Araip.JTL3L	B02:12132486..12137401	0.13	249.01	1915.46154	Seed maturation protein
SSCG81	Araip.DK4JW	B08:11762581..11767634	0.43	239.01	555.837209	Seed biotin-containing protein SBP65 [*Glycine max*]
SSCG82	Aradu.X3CG0	A02:59745890..59751117	0.03	223.67	7455.66667	Nutrient reservoir, putative
SSCG83	Araip.91947	B01:128237278..128244210	0.51	220.45	432.254902	Glutamine synthetase 2
SSCG84	Aradu.XM2MR	A06:101837477..101842574	2.15	215.63	100.293023	Acyl-[acyl-carrier-protein] desaturase
SSCG85	Araip.S3GXY	B09:142678437..142683138	0.63	214.14	339.904762	Fatty acid desaturase 2
SSCG86	Aradu.X9GQ3	A01:102122128..102125802	2.27	212.89	93.784141	Early nodulin related
SSCG87	Araip.H2E95	B09:132437996..132443128	0.13	212.42	1634	Papain family cysteine protease
SSCG88	Aradu.HYY79	A10:2715010..2717854	0.59	209.18	354.542373	Late embryogenesis abundant protein (LEA) group 3 protein
SSCG89	Araip.QXV0R	B03:144454..148756	0.03	199.72	6657.33333	Cell wall protein EXP3
SSCG90	Aradu.FPC2C	A09:111265722..111269712	0.61	187.52	307.409836	Seed maturation protein
SSCG91	Aradu.IZQ3Z	A10:107916715..107927349	0.97	179.33	184.876289	Annexin 8
SSCG92	Araip.84L6B	B09:2158523..2159716	0.41	176.67	430.902439	Late embryogenesis abundant protein (LEA) family protein
SSCG93	Aradu.S2SYE	A08:49306243..49307719	0.07	176.31	2518.71429	Cell wall protein EXP3
SSCG94	Aradu.440M4	A08:37377595..37378358	0.21	174.86	832.666667	Defensin related
SSCG95	Araip.JYP5G	B03:2187447..2191755	1.76	173.95	98.8352273	NAD+:PROTEIN (ADP-ribosyl)-transferase
SSCG96	Araip.9C0MU	B04:3455685..3456542	0	173.83	-	-
SSCG97	Araip.V3XTL	B04:67447309..67448462	0.27	172.23	637.888889	Unknown protein
SSCG98	Aradu.80WBV	A04:1154520..1168569	1.11	160.05	144.189189	Subtilisin-like serine protease 2
SSCG99	Aradu.UJ6Z9	A06:99636586..99638673	0.36	157.81	438.361111	Aldo/keto reductase family oxidoreductase
SSCG100	Aradu.UU57Q	A09:120002036..120004368	0.39	157.26	403.230769	Papain family cysteine protease
SSCG101	Aradu.XGA9X	A07:72920123..72923135	0.14	155.35	1109.64286	PREDICTED: transcription factor HBP-1b(c1)-like isoform X2 [*Glycine max*]
SSCG102	Araip.97QE1	B04:2178764..2181986	0.27	150.15	556.111111	Protein of unknown function
SSCG103	Araip.SP2PF	B09:132209230..132210189	0.18	149.66	831.444444	AWPM-19-like family protein
SSCG104	Aradu.RDK4X	A02:86334489..86339921	0.03	149.58	4986	PREDICTED: probable pectinesterase/pectinesterase inhibitor 36-like [*Glycine max*]
SSCG105	Araip.A9IK4	B04:7236775..7238080	0	137.8	-	-
SSCG106	Araip.BIZ4B	B09:2161015..2161580	0	136.04	-	-
SSCG107	Araip.WF9GZ	B03:128599514..128604032	0.13	134.56	1035.07692	Flowering locus protein T
SSCG108	Araip.X6DZU	B02:99317123..99324724	0.16	134.51	840.6875	PREDICTED: probable pectinesterase/pectinesterase inhibitor 36-like [*Glycine max*]

**Table 2:** GO classification of 337 SSCGs from *A*. *duranensis* and *A*. *ipaensis*.

Ontology	Class	Gene Number
Biological Process	single-organism process	108
	response to stimulus	44
	multicellular organismal process	16
	reproductive process	10
	reproduction	10
	multiorganism process	6
	biological regulation	37
	immune system process	2
	developmental process	17
	signaling	10
	localization	29
	metabolic process	120
	cellular component organization or biogenesis	10
	cellular process	83
Molecular Function	nutrient reservoir activity	23
	molecular function regulator	11
	nucleic acid binding transcription factor activity	14
	antioxidant activity	4
	transporter activity	14
	electron carrier activity	2
	signal transducer activity	2
	catalytic activity	108
	binding	92
Cellular Component	extracellular region	6
	membrane part	29
	cell part	65
	cell	65
	membrane	37
	organelle	44
	organelle part	15
	macromolecular complex	2

### Characterization of the top 108 SSCGs from *A*. *duranensis* and *A*. *ipaensis*

**Fig 1:**
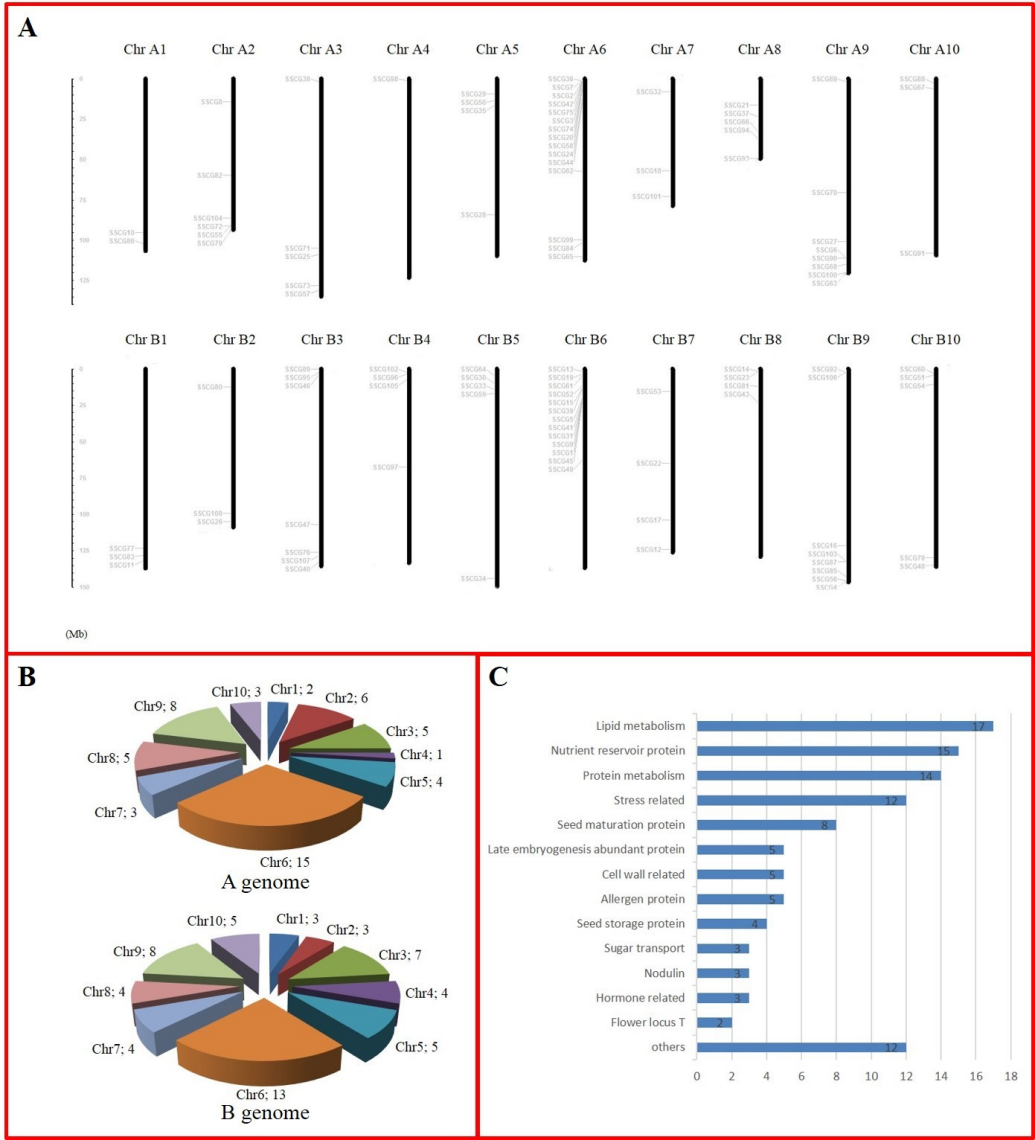
Characterization of the top 108 SSCGs from *A*. *duranensis* and *A*. *ipaensis*. (A) Chromosomal distribution of the 108 SSCGs. The chromosome numbers are shown at the top of each chromosome (black bars). The names on the left of each chromosome correspond to the approximate location of each SSCG. (B) Numbers of SSCGs on each chromosome of *A*. *duranensis* and *A*. *ipaensis*. (C) Functional classification of the 108 SSCGs.

SSPs are usually isolated from seed storage proteins and/or other proteins related to seed development, such as *Brassica napus* Napin, which was isolated from a 2S storage protein [37], indicating that gene characterization may reflect the specificity of its promoter. To predict the activity of their promoters, we therefore characterized the 108 SSCGs. Among the top 108 SSCGs, 96 had putative functions, and 12 had unknown functions. The 96 SSCGs were classified into 14 groups according to their annotations, and 54 of those SSCGs were involved in lipid metabolism and seed maturation or coded for nutrient reservoir proteins, allergens, and seed storage proteins (Fig 1C), which revealed that these top 108 SSCGs might perform functions within peanut seeds.

As shown in Fig 1A, SSCGs were randomly dispersed across 10 chromosomes. In *A*. *duranensis*, chromosome A6 contained the greatest number of SSCGs (15), while chromosome A4 contained the fewest SSCGs (1). In *A*. *ipaensis*, 13 SSCGs were distributed on chromosome B6, whereas only 3 SSCGs were found on chromosomes B1 and B3 (Fig 1B). Several SSCGs were located on the chromosomes in clusters; for example, 6 SSCGs (SSCG2, SSCG3, SSCG7, SSCG36, SSCG42, SSCG75) were within the 1.26–1.8 cM region on chromosome A6 (Fig 1A); functional prediction revealed that these SSCGs encoded nutrient reservoir proteins (Table 1). SSCG14 and SSCG23, both of which coded for seed linoleate 9S-lipoxygenase, were located at the same locus of chromosome B8. These results suggested that these clustered genes might function together in coordination.

In this study, we identified 39 orthologous gene pairs between *A*. *duranensis* and *A*. *ipaensis* based on phylogenetic relationships (S2 Fig, Table 3), among which 36 orthologous gene pairs were found at the syntenic locus on the *A*. *duranensis* and *A*. *ipaensis* chromosomes (Fig 1A, Table 3). The orthologous genes from *A*. *duranensis* and *A*. *ipaensis* exhibited similar functions; for example, both SSCG63 (A9) and SSCG103 (B9) encode the AWPM-19-like family protein, and both SSCG87 (B9) and SSCG100 (A9) encode the papain family cysteine protease (Tables 1 and 3). Although the sequences of some orthologous gene pairs are highly similar, their promoter sequences were sometimes quite different. For example, SSCG43 (Araip.213GN) and SSCG94 (Aradu.440M4) had the same sequence, but their promoter sequences were quite different. Whether the promoters of orthologous gene pairs displayed the same specificity needs to be further determined. The location of 2 SSCGs in the A genome (SSCG21 and SSCG93) did not correspond to the same location of their orthologous genes in the B genome (SSCG12 and SSCG89). Interestingly, SSCG53, located on chromosome B7, had the same sequence as its orthologous gene, SSCG54, on chromosome B10.

**Table 3:** Orthologous gene pairs of the top 108 SSCGs from *A*. *duranensis* and *A*. *ipaensis*.

Gene pair	Chromosome	CDS identity (%)	Protein identity (%)
SSCG1-SSCG5	B06-A06	94.26	87.76
SSCG2-SSCG9	A06-B06	53.81	52.85
SSCG3-SSCG7	B06-A06	90.71	97.17
SSCG4-SSCG6	B09-A09	86.98	85.62
SSCG10-SSCG11	A01-B01	98.91	98.03
SSCG12-SSCG21	B07-A08	87.01	80.41
SSCG13-SSCG44	B06-A06	95.29	93.35
SSCG15-SSCG20	B06-A06	93.15	97.90
SSCG16-SSCG27	B09-A09	98.70	77.69
SSCG18-SSCG22	A07-B07	98.08	97.58
SSCG19-SSCG24	B06-A06	73.78	70.00
SSCG23-SSCG37	B08-A08	96.18	96.06
SSCG26-SSCG72	B02-A02	98.91	73.76
SSCG28-SSCG34	A05-B05	88.57	88.30
SSCG29-SSCG30	A05-B05	93.04	90.00
SSCG31-SSCG42	B06-A06	94.72	94.57
SSCG33-SSCG50	B05-A05	98.89	95.09
SSCG35-SSCG59	A05-B05	96.30	96.10
SSCG36-SSCG45	A06-B06	46.80	46.05
SSCG38-SSCG46	A03-B03	57.65	57.86
SSCG39-SSCG74	B06-A06	90.16	88.11
SSCG40-SSCG57	B03-A03	74.79	52.84
SSCG41-SSCG75	B06-A06	98.47	97.70
SSCG43-SSCG94	B08-A08	100	100
SSCG47-SSCG71	B03-A03	91.57	90.31
SSCG48-SSCG91	B10-A10	94.12	90.37
SSCG49-SSCG62	B06-A06	95.47	93.07
SSCG51-SSCG88	B10-A10	98.55	87.50
SSCG52-SSCG58	B06-A06	98.84	97.30
SSCG53-SSCG54	B07-B10	100	100
SSCG56-SSCG90	B09-A09	98.18	97.66
SSCG63-SSCG103	A09-B09	97.25	98.34
SSCG66-SSCG81	A08-B08	96.92	95.01
SSCG68-SSCG85	A09-B09	86.74	86.04
SSCG69-SSCG92	A09-B09	97.63	96.10
SSCG73-SSCG107	A03-B03	96.80	96.59
SSCG87-SSCG100	B09-A09	98.21	98.11
SSCG89-SSCG93	B03-A08	98.94	98.41
SSCG104-SSCG108	A02-B02	97.16	95.03

### Expression patterns of the top 108 SSCGs

**Fig 2:**
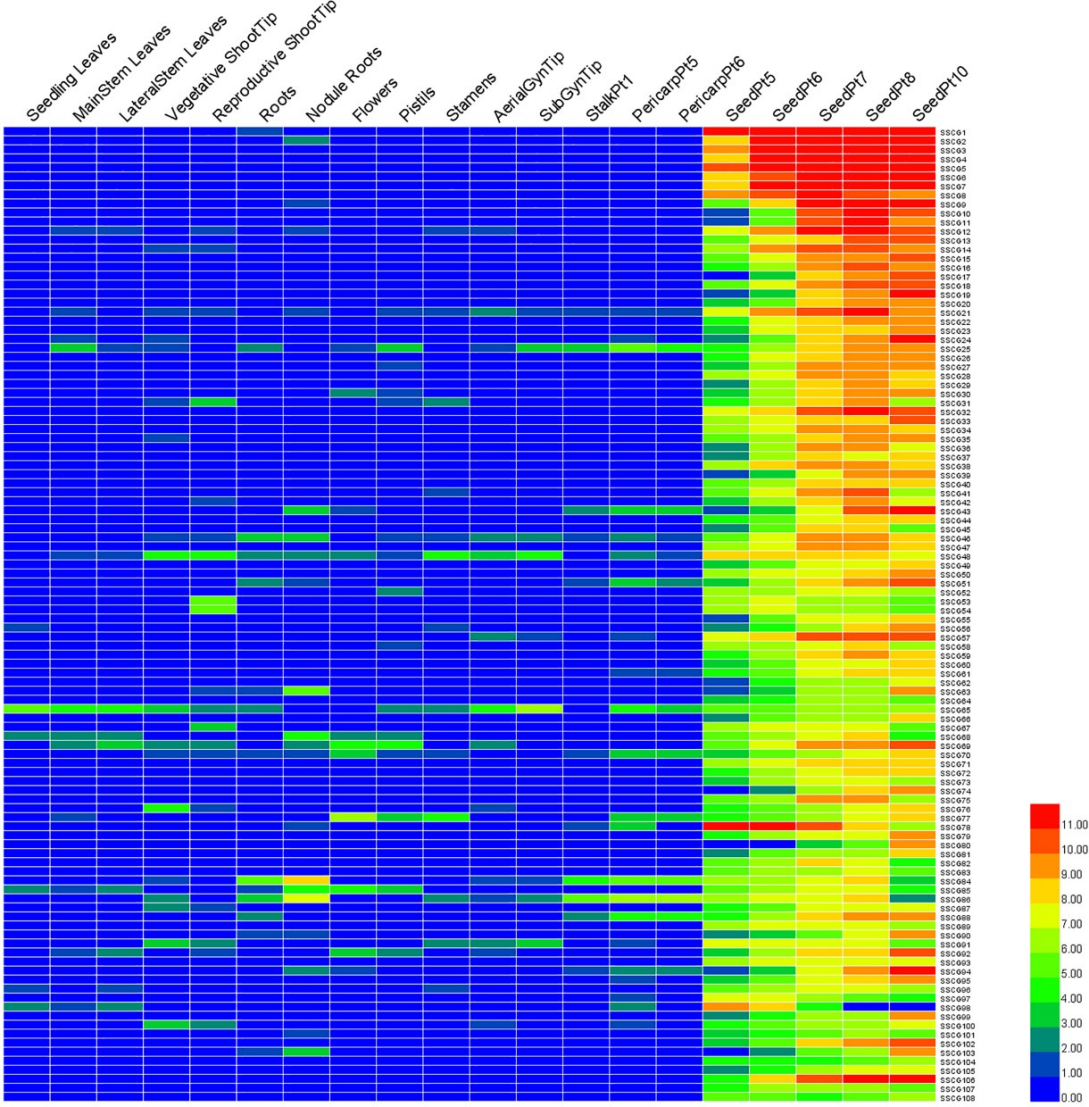
Expression profiles of the top 108 SSCGs in 20 different tissues of *A*. *duranensis* and *A*. *ipaensis*. The FPKM data of 20 distinct tissues for the top 108 SSCGs were retrieved from the work of Clevenger et al. [31]. The FPKM value of each gene was log2-transformed and displayed in the form of heat maps by HemI. The color scale in the lower right represents the relative expression level: green represents a low level, and red indicates a high level. Twenty different tissues are shown on top of the heat map. The SSCGs are listed on the right of the heat map.

To confirm the tissue expression specificity of the top 108 SSCGs, we first analyzed the expression profiles using the expression information provided by Clevenger et al. [31]. The heat map results showed that all the top 108 genes were expressed in the seed; most were expressed only in the seed, whereas the rest were preferentially expressed in the seed (Fig 2). The expression patterns of the orthologous genes from the A and B genomes were similar. For example, SSCG12 and SSCG21 were highly expressed during the Pt6, Pt7, Pt8 and Pt10 seed stages but weakly expressed in other tissues, such as mainstem leaves, the reproductive shoot tip, nodule roots, stamens and the aerial gynophore tip. SSCG78 was expressed in the early seed development stage (SeedPt5-7), while SSCG106 was expressed in the late seed development stage (SeedPt7, 8, 10). Their promoters could be used to express genes at different seed development stages. Notably, SSCG1-12 was extremely highly expressed in the seeds, and specifically, SSCG1 and SSCG6 were abundantly expressed during all five seed development stages (Fig 2). Functional prediction analysis revealed that these SSCGs encoded nutrient reservoir proteins or allergen proteins (Table 1), whose transcripts are considered widely expressed specifically in mature peanut seed [38,39].

**Fig 3:**
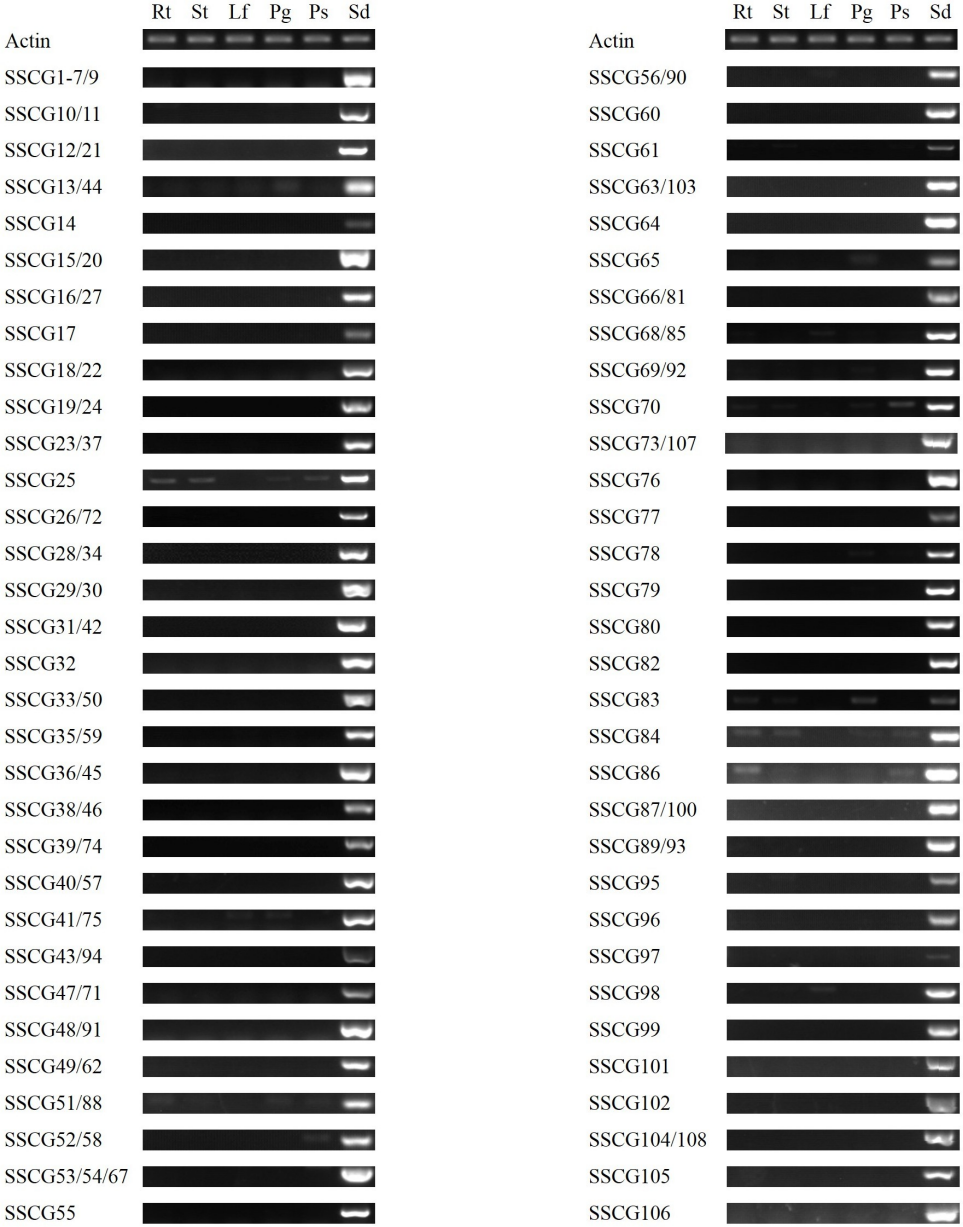
Semiquantitative RT-PCR analysis of the top 108 SSCGs in different tissues of cultivated peanut. Orthologous genes were considered a single gene. Expression patterns were detected in the roots (Rt), stems (St), leaves (Lf), pegs (Pg), pod shells (Ps) and seeds (Sd) using the *Actin* gene as an internal control.

We further examined the tissue expression specificity of the SSCGs in cultivated peanut via semiquantitative RT-PCR. Because the orthologous gene pairs had similar sequences, they were considered a single gene, and to investigate their expression patterns, primers were designed based on their same sequence. As shown in Fig 3, similar to the heat map results, most of these 108 SSCGs were expressed specifically and/or preferentially in the seed. Ninety-four out of the 108 SSCGs were expressed exclusively in the seed, accounting for 87%. Only a few SSCGs (SSCG13, 25, 41, 44, 51, 52, 58, 70, 75, 83, 84, 86, 88, 98) were also weakly expressed in other tissues, such as the roots, stems, pegs, pod shells and leaves.

Overall, based on the expression pattern analysis above, the SSCGs described in this study are potential resources for seed-specific and/or preferential promoter cloning.

### Cis-acting elements in the promoter regions of the top 108 SSCGs

Gene expression specificity was mediated by cis-elements in the promoter region [40,41]. To identify the regulatory cis-elements in the promoter region of SSCGs, we extracted the 2500 bp promoter sequence upstream of the start codon of the top 108 SSCGs. The results showed that there were 92 promoters containing RY REPEAT motifs and 33 promoters containing GCN4 motifs. Thirty-seven promoters contained more than three RY REPEAT motifs, and there were five motifs in SSCG28 (Aradu.DWL7L) and SSCG99 (Aradu.UJ6Z9) and six in SSCG74 (Aradu.9S6MI). Twenty-nine promoter sequences contained both motifs (Table 4). The RY REPEAT (CATGCA) [42] and GCN4 (TGAGTCA) [43,44] motifs are commonly located within seed- and/or embryo-specific promoter sequences. These results implied that most of the promoters of the top 108 SSCGs were seed specific.

**Table 4:** Numbers of two elements, RY REPEAT and GCN4 elements, in the promoter region of the top 108 SSCGs from *A*. *duranensis* and *A*. *ipaensis*.

Gene ID	Gene symbol	RY REPEAT	GCN4
SSCG1	Araip.D61U9	1	0
SSCG2	Aradu.F9TAJ	3	0
SSCG3	Aradu.YGS80	1	0
SSCG4	Araip.T82B5	3	0
SSCG5	Araip.WQE9Q	2	0
SSCG6	Aradu.2H0R0	3	0
SSCG7	Aradu.YBK6Q	4	0
SSCG8	Aradu.B98FL	3	0
SSCG9	Araip.5JB56	4	1
SSCG10	Aradu.I3E1J	3	1
SSCG11	Araip.CF8RS	2	1
SSCG12	Araip.4G9JR	2	1
SSCG13	Araip.16S9Q	4	1
SSCG14	Araip.DH1Z0	4	1
SSCG15	Araip.UPW6L	4	0
SSCG16	Araip.XV8NA	2	0
SSCG17	Araip.930A9	2	1
SSCG18	Aradu.L7CNH	1	0
SSCG19	Araip.GWR7V	0	0
SSCG20	Aradu.P54FB	2	0
SSCG21	Aradu.CPR44	1	1
SSCG22	Araip.TR541	1	0
SSCG23	Araip.E99Y9	3	1
SSCG24	Aradu.A02RY	0	1
SSCG25	Aradu.8NU6I	3	0
SSCG26	Araip.GVB7U	1	0
SSCG27	Aradu.TC8DF	0	1
SSCG28	Aradu.DWL7L	5	1
SSCG29	Aradu.YC8MH	3	1
SSCG30	Araip.MGW36	1	0
SSCG31	Araip.SK1EN	2	0
SSCG32	Aradu.UQE92	3	0
SSCG33	Araip.XXN6R	2	0
SSCG34	Araip.LJX8Z	3	1
SSCG35	Aradu.F1JZ5	1	1
SSCG36	Aradu.1QI16	2	1
SSCG37	Aradu.WX5KP	2	1
SSCG38	Aradu.7S7IW	2	1
SSCG39	Araip.S2F61	4	2
SSCG40	Araip.YX1UI	1	0
SSCG41	Araip.LRG7E	3	0
SSCG42	Aradu.G7AM5	2	0
SSCG43	Araip.213GN	3	0
SSCG44	Aradu.I953D	3	0
SSCG45	Araip.IGC50	2	1
SSCG46	Araip.I9427	2	0
SSCG47	Araip.C23HG	2	0
SSCG48	Araip.Z0Q6Q	2	1
SSCG49	Araip.18621	0	0
SSCG50	Aradu.Y7IVD	3	0
SSCG51	Araip.K8QIE	0	0
SSCG52	Araip.PTL0N	2	0
SSCG53	Araip.8Y88R	2	0
SSCG54	Araip.FWQ8E	2	0
SSCG55	Aradu.QK6K1	3	0
SSCG56	Araip.U0WFW	0	0
SSCG57	Aradu.QV0LR	2	0
SSCG58	Aradu.7HS2D	3	0
SSCG59	Araip.534K5	3	0
SSCG60	Araip.YF7VP	0	0
SSCG61	Araip.55BM4	2	1
SSCG62	Aradu.ZQ8HD	0	1
SSCG63	Aradu.F3KB2	2	0
SSCG64	Araip.Z2VYZ	2	0
SSCG65	Aradu.KPI4B	1	0
SSCG66	Aradu.HX36X	3	1
SSCG67	Aradu.TVV1L	2	0
SSCG68	Aradu.G1YNF	4	0
SSCG69	Aradu.Y6LUX	1	1
SSCG70	Aradu.N27YB	1	0
SSCG71	Aradu.BXD3B	3	0
SSCG72	Aradu.KQ35F	2	1
SSCG73	Aradu.Z8JSI	3	0
SSCG74	Aradu.9S6MI	6	0
SSCG75	Aradu.XDS84	2	0
SSCG76	Araip.27I5U	2	0
SSCG77	Araip.FYJ9U	4	0
SSCG78	Araip.XR8KB	0	0
SSCG79	Aradu.9Z0RX	0	0
SSCG80	Araip.JTL3L	0	0
SSCG81	Araip.DK4JW	0	0
SSCG82	Aradu.X3CG0	2	0
SSCG83	Araip.91947	3	1
SSCG84	Aradu.XM2MR	1	0
SSCG85	Araip.S3GXY	4	0
SSCG86	Aradu.X9GQ3	2	0
SSCG87	Araip.H2E95	2	1
SSCG88	Aradu.HYY79	0	0
SSCG89	Araip.QXV0R	2	1
SSCG90	Aradu.FPC2C	0	0
SSCG91	Aradu.IZQ3Z	2	0
SSCG92	Araip.84L6B	2	0
SSCG93	Aradu.S2SYE	1	1
SSCG94	Aradu.440M4	2	1
SSCG95	Araip.JYP5G	1	1
SSCG96	Araip.9C0MU	1	0
SSCG97	Araip.V3XTL	1	0
SSCG98	Aradu.80WBV	0	0
SSCG99	Aradu.UJ6Z9	5	0
SSCG100	Aradu.UU57Q	3	0
SSCG101	Aradu.XGA9X	2	1
SSCG102	Araip.97QE1	3	0
SSCG103	Araip.SP2PF	2	0
SSCG104	Aradu.RDK4X	3	0
SSCG105	Araip.A9IK4	1	0
SSCG106	Araip.BIZ4B	2	0
SSCG107	Araip.WF9GZ	3	0
SSCG108	Araip.X6DZU	0	0

### Characterization of an SSP

**Fig 4:**
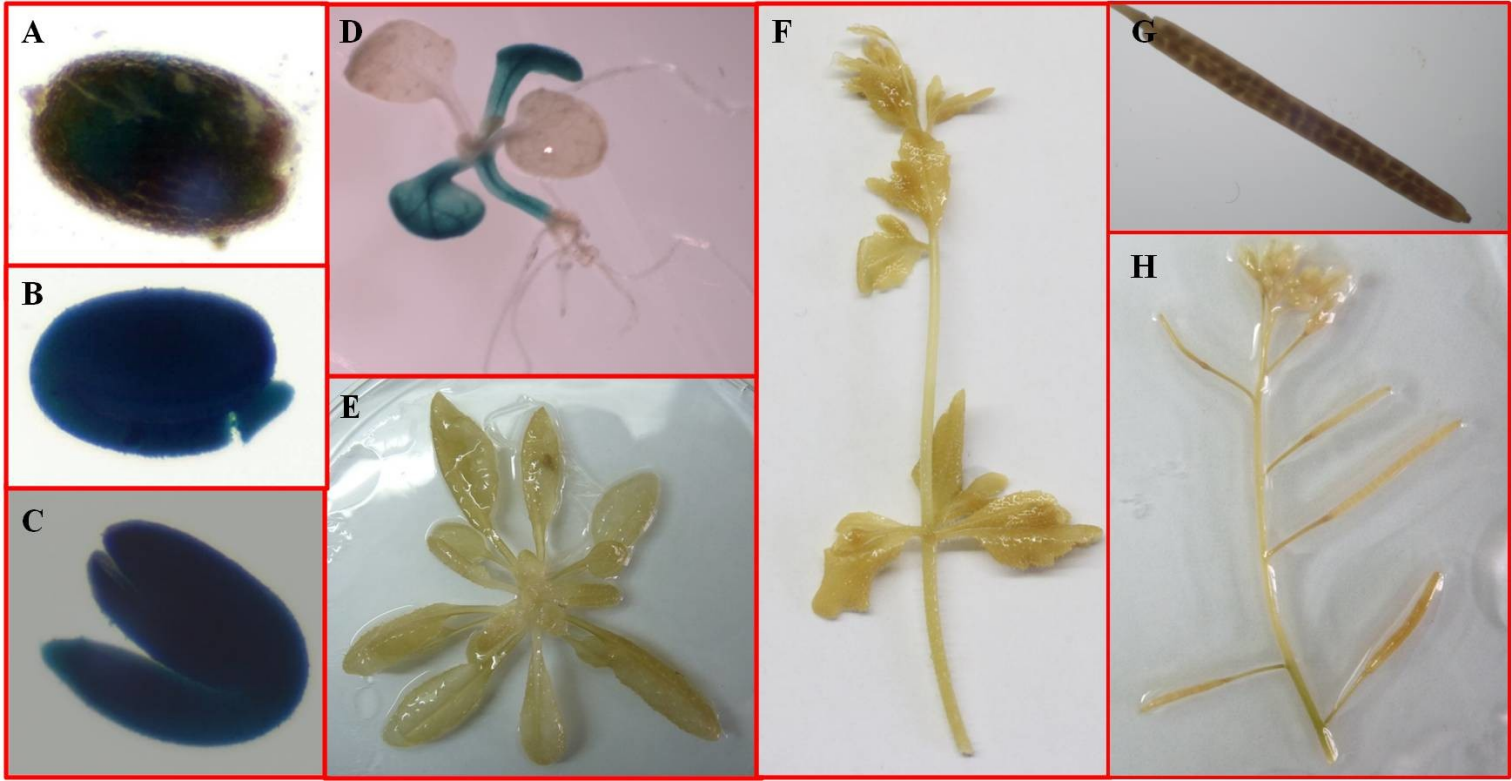
Functional characterization of the putative promoter AHSSP29 in transgenic *Arabidopsis*. (A) Mature seed wrapped in a testa. (B-C) Germinating seed without a testa. (D) Young seedlings with two true leaves. (E) Adult plant. (F) Stem and flower of adult plants.(G-H) Siliques.

To verify promoter tissue specificity, we isolated a 2771 bp promoter fragment (Arachis Hypogaea Seed-Specific Promoter 29, AHSSP29) from the cultivated cultivar peanut ‘Shitouqi’ according to the reference sequence of SSCG29 (Aradu.YC8MH) in its ancestor *A*. *duranensis*. SSCG29 encodes a vicilin-like seed storage protein. Several cis-acting elements, including one GCN4 motif [43,44], two RY REPEATs [42], and three 2SSEEDPROTBANAPAs [45], which commonly exist in SSPs, were detected in the AHSSP29 sequence (Table 5). AHSSP29 was then substituted with the CamV35S promoter in a pBI121 vector to produce a AHSSP29::GUS construct, which was subsequently transformed into *Arabidopsis*. GUS histochemical assays revealed GUS staining in all parts of the seed (Fig 4A–4C), with the exception of the seed testa. GUS staining was hard to observe in seed wrapped in a testa (Fig 4A), while GUS activity was clearly visible in the germinating seed that lacked a testa (Fig 4B and 4C). Definitive staining was also observed in the cotyledons and hypocotyls of the seedlings (Fig 4D), which are components of the seed. No GUS activity was detected in the leaves, stems, flowers, roots and siliques at any time during the plant life cycle (Fig 4E–4H). Nontransformed *Arabidopsis* plants did not display GUS activity in their mature seeds or any parts of the plants. These results suggested that the AHSSP29 promoter was an SSP.

**Table 5:** Putative cis-acting elements in the AHSSP29 promoter sequence.

Element	Sequence	Location	Putative functions
SEF1 MOTIF	ATATTTAWW	-651 (-), -740 (+)	soybean embryo factor 1, found in the 5'-upstream region of the β-conglycinin gene
SEF4 MOTIF	RTTTTTR	-384 (-), -906 (-), -1727 (-), -1923 (+), -2463 (-), -2490 (-), -2541 (+), -2569 (+)	found in soybean 5'-upstream region of the β-conglycinin gene
EBOXBNNAPA	CANNTG	-30 (+), -103 (+), -120 (+), -169 (+), -1652 (+)	E-box of the napA storage protein gene of *Brassica napus*
RY REPEAT	CATGCA	-83 (+), -1782 (+),	required for seed-specific expression
GCN4	TGAGTCA	-443 (-)	required for endosperm-specific expression
2SSEEDPROTBANAPA	CAAACAC	-165 (-), -1069 (+), -2401 (-)	conserved in many storage protein gene promoters; important for high activity of the napA promoter
CANBNNAPA	CNAACAC	-165 (-), -1069 (+), -1881 (+), -2401 (-), -2698 (+)	embryo- and endosperm-specific transcription of the *napin* (storage protein) gene
CAAT-Box	CAAT	-254 (+)	common cis-acting element in promoter and enhancer regions
TATA-Box	TATATA	-67 (+)	core promoter element near -30 of the transcription start site

The symbol ‘+’ or ‘-’ in parentheses represents the DNA strand in which the element is situated.

The negative number indicates the location of elements within AHSSP29.

## Discussion

SSPs are valuable tools for the genetic engineering of seed, especially for seed bioreactor research. Peanut seeds are ideal bioreactors for the production of foreign recombinant proteins and other nutrient metabolites. However, only a few seed-specific and/or seed-preferential promoters have been identified from peanut [17–19,46]. Expressing multiple foreign genes using the same promoters is ill advised [14–16]. Therefore, additional SSPs are urgently needed. In this study, we established an effective method for the genome-scale mining of SSCGs via comparative transcriptome sequencing of a mixture of nonseed tissue and seed tissue. A total of 337 SSCGs were identified, and 108 SSCGs in *A*. *duranensis* and *A*. *ipaensis* were further characterized. At least 94 SSCGs were confirmed via semiquantitative RT-PCR to be expressed specifically in the seed in cultivated peanut, and the rest were preferentially expressed in the seed. This study provided a valuable resource for seed-specific and/or seed-preferential promoter cloning.

Among the 108 identified SSCGs, most functioned in relation to seed development or coded for allergen proteins or storage proteins (Fig 1C, Table 1). For example, SSCG1-7 and SSCG9, which encoded allergen proteins, were homologous genes and were extremely highly expressed according to their FPKM values (Table 1), heat map results (Fig 2) and semiquantitative RT-PCR analysis (Fig 3). Peanut allergen proteins were reported to be expressed exclusively in the seed [39] and accounted for a considerable amount of the total seed protein in peanut [47]. This finding is in accordance with the abundant expression of SSCG1-7 and SSCG9 in the peanut seed. These results indicated that these SSCGs were expressed specifically in the seed, and these SSCGs that were most abundantly expressed were the focus of our subsequent promoter cloning.

Studies have shown that several cis-acting elements in promoter sequences are responsible for mediating gene expression specificity. For example, the cis-acting elements RY REPEAT and GCN4 are conserved among many SSPs [42,43]. These cis-acting elements were also present throughout most of the SSCGs in this study, which implied that the promoters in most of the SSCGs might drive gene expression in a seed-specific manner. Several promoters of these SSCGs have been characterized as SSPs. For example, the promoter of an SSCG5 paralogous gene, which encodes an allergen protein, was isolated and characterized as an SSP [19]. Together with the novel SSP AHSSP29 of SSCG29 (Aradu.YC8MH) identified in this study, which contained 2 RY REPEAT and 1 GCN4 elements, the results indicate that the SSCG mining strategy in this study seemed effective and accurate. Once these promoters are isolated and characterized, they could be widely used for allergen reduction via gene editing technologies and for other research on seed quality improvement.

Geng et al. [48] introduced a method for tissue-specific promoter cloning by comparing expression levels among three tissues: leaves, roots, and seeds. A total of 316 seed-specific candidate transcript assembly contigs (TACs) were identified. In addition, 64.6% of select TACs were expressed exclusively in the seed and not in the leaves, stems, or roots [48]. However, to date, no SSPs have been identified based on these data, which may be attributed to insufficient transcriptome data and the lack of reference genome information. In our study, only two samples were chosen for transcriptome sequencing: seeds from different development stages and a mixture of nonseed tissue from six tissues (including roots, stems, leaves, flowers, pegs, and pod shells). It is much less expensive to sequence the transcriptome of nonseed tissue mixtures than to sequence each individual tissue. Moreover, it becomes simpler and more accurate to screen SSCGs by comparing two samples rather than by comparing numerous samples. Consequently, 337 SSCGs were identified, and 87% of the top 108 SSCGs were expressed exclusively in the seed and not in the five measured tissues (roots, stems, leaves, pegs, and pod shells). These results indicated that additional tissues were necessary as part of the nonseed sample to compare gene expression differences with seed samples. This SSCG information, such as the gene symbols, can be obtained conveniently from Table 1 and S2 Table. Researchers could easily download SSCGs of interest from the PeanutBase website according to this information. With the decreasing transcriptome sequencing cost and the release of the peanut genome, mining tissue-specific genes from peanut via comparative transcriptome sequencing has become a robust approach. For example, contamination with aflatoxin, which is produced in infected peanut seeds by *Aspergillus flavus*, is one of the major problems in peanut production. Given that peanut pericarps are barriers against *A*. *flavus*, pericarp-specific promoters are a good choice for expressing *A*. *flavus*-resistant genes specifically in the pericarp to prevent aflatoxin contamination. Pericarp-specific promoters could be identified by the strategy presented in this study.

## Conclusions

We identified 337 SSCGs by comparative RNA sequencing (RNA-seq) between seed and nonseed tissues. The top 108 SSCGs, according to their FPKM, were characterized, among which 94 were expressed specifically in the seed, and 14 were preferentially expressed in the seed. In addition, a novel SSP, AHSSP29, was functionally characterized. The strategy presented in this study could facilitate the future exploration of tissue-specific promoters in other crop species. Additionally, the SSCGs identified in this work could be widely applied for SSP cloning by other researchers.

## Supporting information

S1 Fig GO annotation of 337 SSCGs identified from *A*.*duranensis* and *A*.*ipaensis*. The Y-axis represents the number of genes in a category.
(JPG)

S2 Fig Phylogenetic relationships of the 108 SSCGs. The phylogenetic tree was constructed with MEGA 6.0 using the NJ method with 1000 bootstrap replicates based on a multiple alignment of 108 SSCGs from *A*.*duranensis* and *A*.*ipaensis*.
(JPG)

S1 Table Summary of the sequence data from Illimina sequencing. (DOCX)

S2 Table List of SSCGs (SSCG109-337) identified from *A*.*duranensis* and *A*.*ipaensis* by comparative transcriptome sequencing. (DOCX)

S3 Table Primers used in this study. (DOCX)
