# Supplementary figures and images for: Genome-wide mining seed-specific candidate genes from peanut for promoter cloning
Source: PLoS One. 2019 Mar 28;14(3):e0214025. doi: 10.1371/journal.pone.0214025 (PMC6438489; doi:10.1371/journal.pone.0214025)

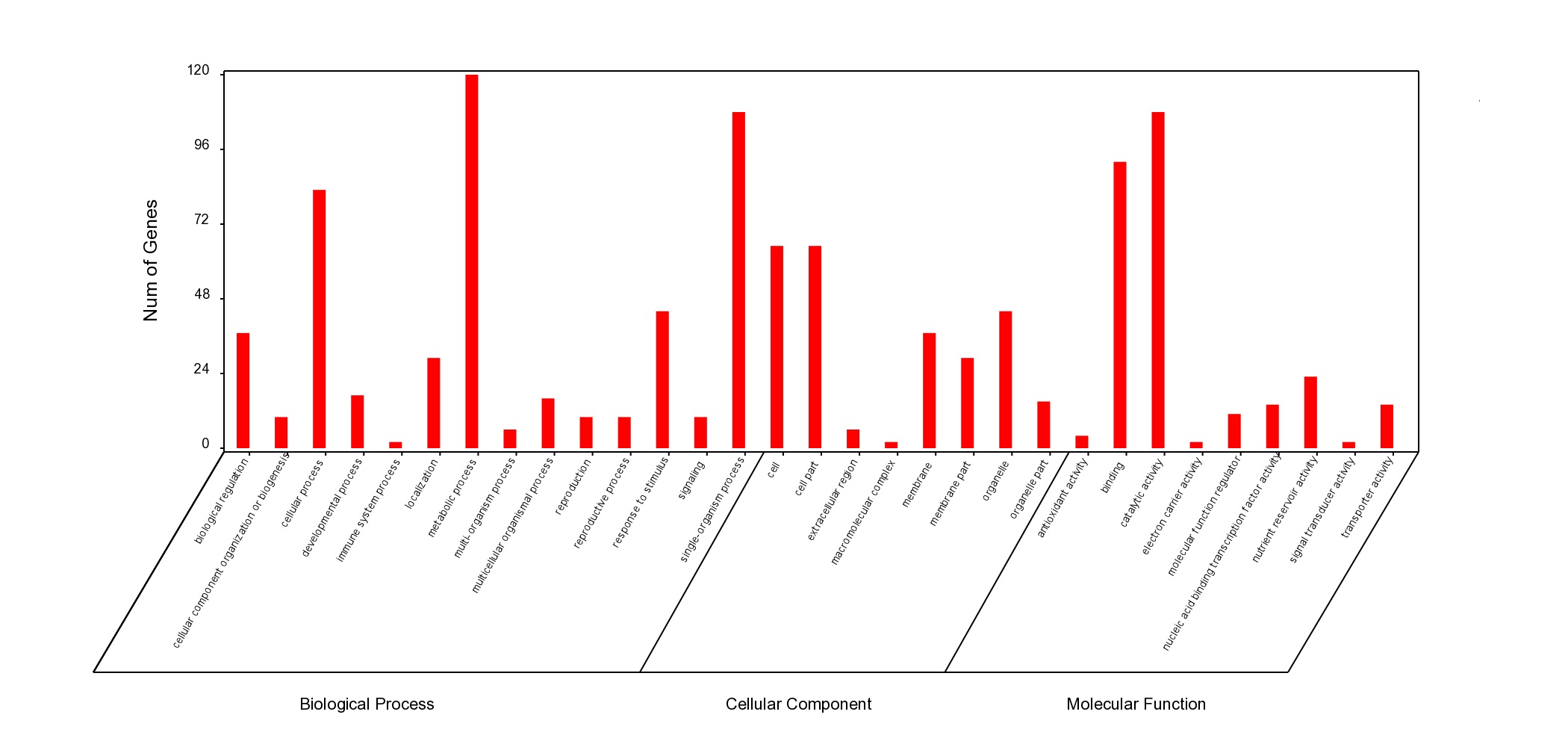

Supplement: S1 Fig — The Y-axis represents the number of genes in a category. (JPG) [file pone.0214025.s001.jpg]

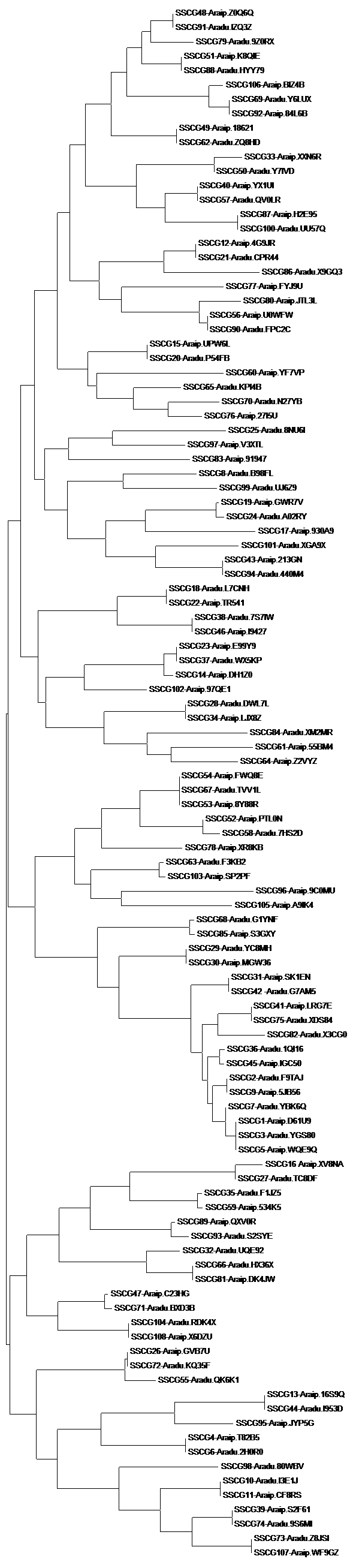

Supplement: S2 Fig — The phylogenetic tree was constructed with MEGA 6.0 using the NJ method with 1000 bootstrap replicates based on a multiple alignment of 108 SSCGs from A.duranensis and A.ipaensis. (JPG) [file pone.0214025.s002.jpg]
