# Supplementary material for: Genome-wide mining seed-specific candidate genes from peanut for promoter cloning
Source: PLoS One. 2019 Mar 28;14(3):e0214025. doi: 10.1371/journal.pone.0214025 (PMC6438489; doi:10.1371/journal.pone.0214025)
Supplement: S1 Table — (DOCX) [file pone.0214025.s003.docx]

**S1 Table. Summary of the sequence data from Illimina sequencing**

| Sample | Raw reads | HQ Clean reads | Clean bases (Gb) | Error (%) | Q20 (%) | Q30 (%) | GC content (%) |
| --- | --- | --- | --- | --- | --- | --- | --- |
| I (seed) | 76794912 | 75373360 | 11.25 | 0.00 | 93.76% | 85.67% | 47.52% |
| II (non-seed) | 78930134 | 77816124 | 11.61 | 0.00 | 94.23% | 86.60% | 45.51% |
