# Supplementary material for: Genome-wide mining seed-specific candidate genes from peanut for promoter cloning
Source: PLoS One. 2019 Mar 28;14(3):e0214025. doi: 10.1371/journal.pone.0214025 (PMC6438489; doi:10.1371/journal.pone.0214025)
Supplement: S2 Table — (DOCX) [file pone.0214025.s004.docx]

**S2 Table. List of SSCGs (SSCG109-337) identified from *A.duranensis* and *A.ipaensis* by comparative transcriptome sequencing.**

| ID | Gene symbols | Non-seed PFKM | Seed PFKM | Seed PFKM/Non-seed PFKM | Putative functions |
| --- | --- | --- | --- | --- | --- |
| SSCG109 | Aradu.56WQE | 0.45 | 133.67 | 297.044444 | Unknown protein |
| SSCG110 | Araip.3K0PR | 0 | 132.63 | - | PREDICTED: B3 domain-containing transcription factor FUS3-like [*Glycine max*] |
| SSCG111 | Araip.1L9GW | 1.99 | 132.59 | 66.6281407 | early nodulin-related |
| SSCG112 | Aradu.9L8G9 | 0.12 | 128.77 | 1073.08333 | Protein of unknown function (DUF1264) |
| SSCG113 | Araip.YQ2F8 | 0.1 | 124.18 | 1241.8 | Reticulon family protein |
| SSCG114 | Aradu.HC115 | 0.06 | 122.07 | 2034.5 | PREDICTED: probable galactinol--sucrose galactosyltransferase 2-like isoform X2 [*Glycine max*] |
| SSCG115 | Aradu.18FWJ | 2.25 | 120.46 | 53.5377778 | Non-specific lipid-transfer protein |
| SSCG116 | Araip.TWH1T | 0 | 120.35 | - | Glycoprotein membrane precursor GPI-anchored |
| SSCG117 | Araip.VM6WY | 2.18 | 119.71 | 54.912844 | Gibberellin-regulated family protein |
| SSCG118 | Aradu.24LGE | 0.13 | 118.98 | 915.230769 | SNF1-related kinase regulatory subunit gamma 1 |
| SSCG119 | Araip.UUV1V | 0 | 114.63 | - | Non-specific lipid-transfer protein |
| SSCG120 | Aradu.T9WVT | 0.15 | 114.4 | 762.666667 | Protein of unknown function (DUF1264) |
| SSCG121 | Araip.BL4R3 | 1.04 | 111.92 | 107.615385 | cytochrome P450 |
| SSCG122 | Araip.JLI3V | 0.06 | 110.31 | 1838.5 | Lactoylglutathione lyase / glyoxalase I family protein |
| SSCG123 | Araip.1K5KM | 0.28 | 107.95 | 385.535714 | triacylglycerol lipase |
| SSCG124 | Araip.9R47S | 0.22 | 105.43 | 479.227273 | Unknown protein |
| SSCG125 | Araip.3PA39 | 1.32 | 104.6 | 79.2424242 | potato type II proteinase inhibitor family protein |
| SSCG126 | Aradu.WHM45 | 0.12 | 103.66 | 863.833333 | Late embryogenesis abundant protein group 3 protein |
| SSCG127 | Aradu.TS7UQ | 0.19 | 103.48 | 544.631579 | late embryogenesis abundant protein |
| SSCG128 | Araip.H76K9 | 0.32 | 102.67 | 320.84375 | short-chain dehydrogenase-reductase B |
| SSCG129 | Araip.PU48P | 0.06 | 102.63 | 1710.5 | PREDICTED: B3 domain-containing transcription factor ABI3-like isoform X1 [*Glycine max*] |
| SSCG130 | Aradu.14IQ9 | 0 | 102.62 | - | Unknown protein |
| SSCG131 | Araip.7T7AZ | 0.03 | 101.68 | 3389.33333 | PREDICTED: transcription factor HBP-1b(c1)-like [*Glycine max*] |
| SSCG132 | Aradu.EI7Y8 | 0.03 | 94.74 | 3158 | PREDICTED: B3 domain-containing transcription factor FUS3-like [*Glycine max*] |
| SSCG133 | Aradu.13SFN | 0.32 | 94.35 | 294.84375 | GDSL-like Lipase/Acylhydrolase superfamily protein |
| SSCG134 | Araip.A4RNH | 0.18 | 87.95 | 488.611111 | Unknown protein |
| SSCG135 | Aradu.U3F37 | 1.72 | 87.88 | 51.0930233 | PREDICTED: uncharacterized protein LOC100819278 [*Glycine max*] |
| SSCG136 | Aradu.9P5WT | 0 | 86.87 | - | PREDICTED: ethylene-responsive transcription factor 7-like [*Glycine max*] |
| SSCG137 | Araip.B9V1J | 1.02 | 86.56 | 84.8627451 | PREDICTED: uncharacterized protein LOC100791441 [*Glycine max*] |
| SSCG138 | Aradu.64BNM | 0.3 | 83.65 | 278.833333 | short-chain dehydrogenase-reductase B |
| SSCG139 | Araip.J17JT | 0 | 83.57 | - | Late embryogenesis abundant protein (LEA) family protein |
| SSCG140 | Aradu.HE28Y | 0.61 | 82.94 | 135.967213 | PREDICTED: probable 2-oxoglutarate/Fe(II)-dependent dioxygenase [*Glycine max*] |
| SSCG141 | Araip.KJK12 | 1.34 | 81.05 | 60.4850746 | PREDICTED: ADP,ATP carrier protein 3, mitochondrial-like [*Glycine max*] |
| SSCG142 | Aradu.7SA2L | 0.05 | 79.52 | 1590.4 | Lactoylglutathione lyase / glyoxalase I family protein |
| SSCG143 | Araip.UB73G | 0 | 78.71 | - | Unknown protein |
| SSCG144 | Aradu.Q97KS | 0.75 | 78.34 | 104.453333 | ER lumen protein retaining receptor family protein |
| SSCG145 | Aradu.L99DH | 0.1 | 76.72 | 767.2 | Unknown protein |
| SSCG146 | Araip.43I77 | 0.08 | 76.54 | 956.75 | lysosomal pro-X carboxypeptidase-like protein |
| SSCG147 | Aradu.LAI2T | 0.14 | 76.3 | 545 | triacylglycerol lipase |
| SSCG148 | Araip.337EV | 0.06 | 76.19 | 1269.83333 | 1-deoxy-D-xylulose 5-phosphate synthase 1 |
| SSCG149 | Araip.EW35C | 0.03 | 75.02 | 2500.66667 | Putative adipose-regulatory protein (Seipin) |
| SSCG150 | Aradu.B4D1B | 0 | 74.29 | - | Glycoprotein membrane precursor GPI-anchored |
| SSCG151 | Aradu.TN44Y | 0.17 | 72.48 | 426.352941 | PREDICTED: gibberellin 20 oxidase 2-like [*Glycine max*] |
| SSCG152 | Aradu.4V3WP | 0.07 | 72.3 | 1032.85714 | PREDICTED: blue copper protein-like [*Glycine max*] |
| SSCG153 | Araip.SY4A0 | 0 | 71.96 | - | Beta-1,3-N-Acetylglucosaminyltransferase family protein |
| SSCG154 | Araip.SVT7Q | 0.15 | 69.47 | 463.133333 | isocitrate lyase |
| SSCG155 | Aradu.60NUI | 0.14 | 69.03 | 493.071429 | PEBP (phosphatidylethanolamine-binding protein) family protein LENGTH=162 |
| SSCG156 | Araip.6N7QF | 0.33 | 68.59 | 207.848485 | PREDICTED: probable 2-oxoglutarate/Fe(II)-dependent dioxygenase-like [*Glycine max*] |
| SSCG157 | Araip.N419E | 0.1 | 66.05 | 660.5 | late embryogenesis abundant protein, putative / LEA protein, putative |
| SSCG158 | Araip.E5JBL | 0.73 | 65.81 | 90.1506849 | P-loop containing nucleoside triphosphate hydrolases superfamily protein |
| SSCG159 | Aradu.TZ6F8 | 0.03 | 63.99 | 2133 | PREDICTED: myb-like protein X-like [*Glycine max*] |
| SSCG160 | Araip.8J30Z | 0 | 63.76 | - | Na+/H+ (sodium hydrogen) exchanger 3 |
| SSCG161 | Aradu.PYT5N | 0.17 | 62.6 | 368.235294 | Bifunctional inhibitor/lipid-transfer protein/seed storage 2S albumin superfamily protein |
| SSCG162 | Aradu.Y8M1J | 0 | 57.42 | - | UDP-Glycosyltransferase superfamily protein |
| SSCG163 | Araip.46XVA | 0.63 | 57.36 | 91.047619 | ferritin 4 |
| SSCG164 | Araip.RCX2B | 0.02 | 55.68 | 2784 | Late embryogenesis abundant protein (LEA) family protein |
| SSCG165 | Aradu.38JU0 | 0.07 | 54.65 | 780.714286 | basic helix-loop-helix (bHLH) DNA-binding superfamily protein |
| SSCG166 | Aradu.61J4L | 0.03 | 54.61 | 1820.33333 | Late embryogenesis abundant protein (LEA) family protein |
| SSCG167 | Araip.EV8J4 | 0.7 | 53.88 | 76.9714286 | myo-inositol oxygenase 5 |
| SSCG168 | Aradu.Q1F6L | 0 | 53.84 | - | Mitochondrial import inner membrane translocase subunit Tim17/Tim22/Tim23 family protein |
| SSCG169 | Araip.D6V1K | 0.4 | 53.06 | 132.65 | terpene synthase 04 |
| SSCG170 | Aradu.LBR45 | 0.02 | 52.73 | 2636.5 | PREDICTED: B3 domain-containing transcription factor ABI3-like isoform X1 [*Glycine max*] |
| SSCG171 | Araip.S13N3 | 0.05 | 52.49 | 1049.8 | Late embryogenesis abundant protein, group 1 protein |
| SSCG172 | Araip.1Y1N6 | 0.02 | 51.67 | 2583.5 | cytochrome P450, family 88, subfamily A, polypeptide 3 |
| SSCG173 | Aradu.TBA51 | 0 | 51.57 | - | Late embryogenesis abundant protein, group 1 protein |
| SSCG174 | Aradu.G3TP5 | 0.09 | 51.45 | 571.666667 | Beta-1,3-N-Acetylglucosaminyltransferase family protein |
| SSCG175 | Araip.UQ6JK | 0 | 51.19 | - | Eukaryotic aspartyl protease family protein |
| SSCG176 | Araip.H0K9H | 0 | 50.85 | - | Histone superfamily protein |
| SSCG177 | Aradu.0KG04 | 0.05 | 50.77 | 1015.4 | isocitrate lyase |
| SSCG178 | Araip.MZ5SZ | 0.17 | 50.4 | 296.470588 | PREDICTED: glycine-rich RNA-binding, abscisic acid-inducible protein-like [*Glycine max*] |
| SSCG179 | Araip.YE1CZ | 0.1 | 50.05 | 500.5 | B3 DNA-binding domain protein |
| SSCG180 | Araip.5XD8H | 0 | 49.05 | - | PREDICTED: gibberellin 20 oxidase 2-like [*Glycine max*] |
| SSCG181 | Aradu.XR48Z | 0 | 47.87 | - | PREDICTED: organ-specific protein S2-like [*Glycine max*] |
| SSCG182 | Aradu.DC866 | 0.86 | 47.45 | 55.1744186 | AWPM-19-like family protein |
| SSCG183 | Araip.1SK7V | 0.4 | 47.42 | 118.55 | PREDICTED: uncharacterized protein LOC100819278 [*Glycine max*] |
| SSCG184 | Aradu.3G22S | 0.04 | 46.29 | 1157.25 | Seed maturation protein LENGTH=262 |
| SSCG185 | Aradu.BFJ1U | 0.21 | 44.91 | 213.857143 | Unknown protein |
| SSCG186 | Aradu.S7WZG | 0.11 | 43.13 | 392.090909 | Mitochondrial import inner membrane translocase subunit Tim17/Tim22/Tim23 family protein |
| SSCG187 | Araip.XH7KH | 0.46 | 43.04 | 93.5652174 | Cell wall protein Exp1 |
| SSCG188 | Aradu.LY749 | 0 | 42.66 | - | Na+/H+ (sodium hydrogen) exchanger 3 |
| SSCG189 | Araip.X37CH | 0.03 | 42.42 | 1414 | PREDICTED: isoflavone reductase-like protein-like [*Glycine max*] |
| SSCG190 | Aradu.D1YQE | 0.12 | 41.94 | 349.5 | DNAJ-like 20 |
| SSCG191 | Aradu.CKL4Y | 0.54 | 41.78 | 77.3703704 | glyoxal oxidase-related protein |
| SSCG192 | Aradu.RR770 | 0.69 | 41.5 | 60.1449275 | PREDICTED: ADP,ATP carrier protein 3, mitochondrial-like [*Glycine* *max*] |
| SSCG193 | Araip.T0VRT | 0.08 | 41.1 | 513.75 | Cellulase (glycosyl hydrolase family 5) protein |
| SSCG194 | Araip.ZKD8M | 0.12 | 41.01 | 341.75 | dessication-induced 1VOC superfamily protein |
| SSCG195 | Aradu.FI39R | 0 | 40.88 | - | PREDICTED: gibberellin 20 oxidase 2-like [*Glycine* *max*] |
| SSCG196 | Araip.UE2UP | 0.04 | 40.63 | 1015.75 | UDP-Glycosyltransferase superfamily protein |
| SSCG197 | Araip.PAL72 | 0.14 | 39.5 | 282.142857 | PREDICTED: blue copper protein-like [*Glycine* *max*] |
| SSCG198 | Aradu.CM57L | 0.31 | 38.93 | 125.580645 | potato type II proteinase inhibitor family protein |
| SSCG199 | Aradu.ISR38 | 0 | 38.69 | - | Unknown protein |
| SSCG200 | Aradu.K0L5G | 0.03 | 38.46 | 1282 | PREDICTED: uncharacterized vacuolar membrane protein YML018C-like isoform X1 [*Glycine* *max*] |
| SSCG201 | Araip.MCM9B | 0 | 38.07 | - | LCR related |
| SSCG202 | Aradu.A3BTB | 0.41 | 37.01 | 90.2682927 | hypothetical protein |
| SSCG203 | Aradu.NH6FA | 0.05 | 36.79 | 735.8 | YABBY transcription factor |
| SSCG204 | Araip.YU9KT | 0.16 | 36.64 | 229 | Unknown protein |
| SSCG205 | Aradu.9B7V9 | 0 | 36.56 | - | Unknown protein |
| SSCG206 | Aradu.FT5QT | 0.08 | 36.4 | 455 | lysosomal pro-X carboxypeptidase-like protein |
| SSCG207 | Araip.FAG7U | 0.02 | 35.89 | 1794.5 | nutrient reservoir protein, putative |
| SSCG208 | Aradu.418U8 | 0.68 | 35.87 | 52.75 | PREDICTED: ent-copalyl diphosphate synthase, chloroplastic-like isoform X1 [*Glycine* *max*] |
| SSCG209 | Araip.4F7TS | 0.4 | 35.75 | 89.375 | PREDICTED: probable 2-oxoglutarate/Fe(II)-dependent dioxygenase [*Glycine* *max*] |
| SSCG210 | Araip.ZEV2T | 0.56 | 35.71 | 63.7678571 | Xyloglucan endotransglucosylase/hydrolase family protein |
| SSCG211 | Aradu.BUI3V | 0.05 | 35.65 | 713 | cytochrome P450, family 88, subfamily A, polypeptide 3 |
| SSCG212 | Araip.3R88A | 0 | 35.29 | - | PREDICTED: anthocyanin 5-aromatic acyltransferase-like [*Glycine max*] |
| SSCG213 | Araip.T7PRK | 0.18 | 35.27 | 195.944444 | Mitochondrial import inner membrane translocase subunit Tim17/Tim22/Tim23 family protein |
| SSCG214 | Araip.D7DHM | 0.7 | 35.14 | 50.2 | hypothetical protein |
| SSCG215 | Araip.GJZ9T | 0.16 | 34.8 | 217.5 | hypothetical protein |
| SSCG216 | Aradu.37CBG | 0 | 34.42 | - | Bifunctional inhibitor/lipid-transfer protein/seed storage 2S albumin superfamily protein |
| SSCG217 | Araip.3237Z | 0 | 34.35 | - | Gibberellin-regulated family protein |
| SSCG218 | Araip.I3LJK | 0.02 | 34.06 | 1703 | B3 DNA-binding domain protein |
| SSCG219 | Aradu.UD1B0 | 0.02 | 33.12 | 1656 | PREDICTED: B3 domain-containing transcription factor ABI3-like isoform X1 [*Glycine max*] |
| SSCG220 | Araip.X3UTX | 0.04 | 32.71 | 817.75 | abscisic acid responsive element-binding factor 1 |
| SSCG221 | Aradu.B5Z5V | 0 | 31.86 | - | Unknown protein |
| SSCG222 | Aradu.AC956 | 0.24 | 31.69 | 132.041667 | seed linoleate 9S-lipoxygenase |
| SSCG223 | Aradu.Q6TEP | 0.14 | 31.67 | 226.214286 | B3 DNA-binding domain protein |
| SSCG224 | Aradu.W4RCV | 0 | 31.36 | - | ferritin 4 |
| SSCG225 | Aradu.401HT | 0.6 | 31.03 | 51.7166667 | hypothetical protein |
| SSCG226 | Araip.62ZBV | 0 | 30.74 | - | Unknown protein |
| SSCG227 | Araip.21MMH | 0.35 | 30.66 | 87.6 | SNF1-related protein kinase regulatory subunit gamma 1 |
| SSCG228 | Aradu.H6MJA | 0.25 | 30.45 | 121.8 | PREDICTED: uncharacterized protein LOC100791441 [*Glycine max*] |
| SSCG229 | Aradu.X0F3K | 0.03 | 30.08 | 1002.66667 | abscisic acid responsive element-binding factor 1 |
| SSCG230 | Araip.MM2AH | 0.08 | 29.55 | 369.375 | Unknown protein |
| SSCG231 | Aradu.JK5AR | 0 | 29.43 | - | Unknown protein |
| SSCG232 | Araip.32F9I | 0 | 29.29 | - | B3 DNA-binding domain protein |
| SSCG233 | Araip.DTX6D | 0 | 28.68 | - | Lipid transfer protein |
| SSCG234 | Araip.PN2Q6 | 0 | 28.41 | - | PREDICTED: uncharacterized vacuolar membrane protein YML018C-like isoform X1 [*Glycine max*] |
| SSCG235 | Aradu.XUD70 | 0.03 | 28.34 | 944.666667 | PREDICTED: protein SCARECROW-like [*Glycine* *max*] |
| SSCG236 | Aradu.Q0MV7 | 0.09 | 27.15 | 301.666667 | PREDICTED: cell wall / vacuolar inhibitor of fructosidase 1-like [*Glycine max*] |
| SSCG237 | Aradu.DU749 | 0 | 26.82 | - | Unknown protein |
| SSCG238 | Araip.MSY72 | 0.12 | 26.7 | 222.5 | PREDICTED: low-temperature-induced 65 kDa protein-like [*Glycine max*] |
| SSCG239 | Aradu.94J1A | 0 | 26.18 | - | PREDICTED: cysteine proteinase inhibitor 4-like [*Glycine max*] |
| SSCG240 | Araip.B6Q3S | 0.09 | 26.13 | 290.333333 | GDSL-like Lipase/Acylhydrolase superfamily protein |
| SSCG241 | Aradu.Y7L8M | 0 | 26.12 | - | leguminosin group486 secreted peptide |
| SSCG242 | Araip.G4XXT | 0 | 25.53 | - | PREDICTED: uncharacterized protein LOC100778708 isoform X3 [*Glycine max*] |
| SSCG243 | Aradu.H9GMI | 0 | 25.28 | - | Putative adipose-regulatory protein (Seipin) |
| SSCG244 | Araip.EG5GC | 0.04 | 24.99 | 624.75 | Peroxidase superfamily protein |
| SSCG245 | Araip.8M2Q8 | 0.26 | 24.87 | 95.6538462 | Glucose-methanol-choline (GMC) oxidoreductase family protein |
| SSCG246 | Araip.SK0HG | 0.12 | 24.8 | 206.666667 | PREDICTED: probable pectinesterase/pectinesterase inhibitor 40-like [*Glycine max*] |
| SSCG247 | Aradu.JZZ9Y | 0 | 24.59 | - | PREDICTED: seed biotin-containing protein SBP65-like [Glycine max] |
| SSCG248 | Araip.WYD1G | 0.07 | 24.29 | 347 | NAD(P)-binding Rossmann-fold superfamily protein |
| SSCG249 | Araip.XI3RH | 0 | 24.18 | - | Unknown protein |
| SSCG250 | Araip.WM0UU | 0.35 | 23.94 | 68.4 | Cytochrome P450 superfamily protein |
| SSCG251 | Araip.V760P | 0.08 | 23.55 | 294.375 | lipid transfer protein 12 |
| SSCG252 | Aradu.WW65Q | 0.38 | 23.4 | 61.5789474 | PREDICTED: ninja-family protein AFP3-like isoform X3 [*Glycine max*] |
| SSCG253 | Araip.W4FSU | 0.34 | 22.8 | 67.0588235 | PREDICTED: cysteine proteinase inhibitor 4-like [*Glycine max*] |
| SSCG254 | Aradu.40JMZ | 0.23 | 22.16 | 96.3478261 | 3-ketoacyl-CoA synthase 19 |
| SSCG255 | Araip.R5S93 | 0 | 21.41 | - | WRKY family transcription factor family protein |
| SSCG256 | Aradu.WQL62 | 0 | 21.18 | - | PREDICTED: isoflavone reductase-like protein-like [*Glycine* *max*] |
| SSCG257 | Aradu.FYC2C | 0 | 21.17 | - | Unknown protein |
| SSCG258 | Aradu.C824H | 0.4 | 21.09 | 52.725 | F-box/RNI-like superfamily protein |
| SSCG259 | Aradu.UG0RV | 0.1 | 20.82 | 208.2 | MADS-box transcription factor family protein |
| SSCG260 | Araip.N2800 | 0.12 | 20.71 | 172.583333 | PREDICTED: seed biotin-containing protein SBP65-like [*Glycine* *max*] |
| SSCG261 | Araip.837LF | 0 | 20.15 | - | Adenine nucleotide alpha hydrolases-like superfamily protein |
| SSCG262 | Araip.5MC2N | 0.38 | 19.65 | 51.7105263 | 3-hydroxyacyl-[acyl-carrier-protein] dehydratase FabZ |
| SSCG263 | Araip.RCN03 | 0.15 | 19.58 | 130.533333 | unknown protein |
| SSCG264 | Aradu.75AIB | 0.1 | 19.14 | 191.4 | ninja-family protein |
| SSCG265 | Araip.U125N | 0.07 | 18.93 | 270.428571 | Unknown protein |
| SSCG266 | Aradu.EJI6Q | 0 | 18.84 | - | PREDICTED: AP2-like ethylene-responsive transcription factor ANT-like [*Glycine* *max*] |
| SSCG267 | Araip.PH0UI | 0.36 | 18.81 | 52.25 | \| transmembrane protein, putative |
| SSCG268 | Araip.286RN | 0 | 18.23 | - | Unknown protein |
| SSCG269 | Araip.B9EY6 | 0 | 17.96 | - | Unknown protein |
| SSCG270 | Araip.7DT5A | 0.03 | 17.93 | 597.666667 | Cytochrome P450 superfamily protein |
| SSCG271 | Aradu.885IZ | 0.09 | 17.86 | 198.444444 | Unknown protein |
| SSCG272 | Aradu.0D9XJ | 0.02 | 17.81 | 890.5 | Peroxidase superfamily protein |
| SSCG273 | Aradu.88K4F | 0 | 17.73 | - | RING finger and CHY zinc finger protein |
| SSCG274 | Araip.X6JXD | 0 | 17.56 | - | B3 DNA-binding domain protein |
| SSCG275 | Aradu.YHW10 | 0.18 | 17.45 | 96.9444444 | Cellulase (glycosyl hydrolase family 5) protein |
| SSCG276 | Araip.ZCR91 | 0.17 | 17.37 | 102.176471 | MADS-box transcription factor family protein |
| SSCG277 | Aradu.79876 | 0.16 | 17.22 | 107.625 | PREDICTED: probable glycosyltransferase At5g03795-like [*Glycine* *max*] |
| SSCG278 | Aradu.19YSM | 0 | 17.12 | - | transferring glycosyl group transferase |
| SSCG279 | Aradu.D0CN9 | 0.02 | 16.57 | 828.5 | laccase 14 |
| SSCG280 | Aradu.MK8Z3 | 0.03 | 16.53 | 551 | Cytochrome P450 superfamily protein |
| SSCG281 | Aradu.TKK2L | 0 | 16.17 | - | B3 DNA-binding domain protein |
| SSCG282 | Aradu.IMK3L | 0.15 | 16.11 | 107.4 | ribonuclease H |
| SSCG283 | Araip.ZDE4L | 0.2 | 15.88 | 79.4 | PREDICTED: cell wall / vacuolar inhibitor of fructosidase 1-like [*Glycine* *max*] |
| SSCG284 | Aradu.7V43S | 0.31 | 15.87 | 51.1935484 | transcription elongation factor-like protein |
| SSCG285 | Aradu.B01FN | 0 | 15.79 | - | PREDICTED: ethylene-responsive transcription factor 4-like [*Glycine* *max*] |
| SSCG286 | Araip.0Y9HM | 0.16 | 15.78 | 98.625 | Protein kinase superfamily protein |
| SSCG287 | Araip.DE4JR | 0.03 | 15.64 | 521.333333 | PREDICTED: adiponectin receptor protein 2-like isoform X3 [*Glycine* *max*] |
| SSCG288 | Aradu.TJ4B5 | 0.26 | 15.53 | 59.7307692 | B3 DNA-binding domain protein |
| SSCG289 | Araip.GX9NA | 0 | 15.51 | - | leguminosin group486 secreted peptide |
| SSCG290 | Araip.XY52C | 0 | 15.46 | - | transferring glycosyl group transferase |
| SSCG291 | Aradu.WY1XI | 0 | 15.36 | - | hypothetical protein |
| SSCG292 | Aradu.J8XT5 | 0.23 | 14.92 | 64.8695652 | Unknown protein |
| SSCG293 | Araip.TS4S8 | 0 | 14.65 | - | Unknown protein |
| SSCG294 | Aradu.KQX01 | 0.08 | 14.32 | 179 | aldolase like |
| SSCG295 | Araip.RZ2UN | 0.02 | 14.22 | 711 | alpha-L-arabinofuranosidase 1 |
| SSCG296 | Araip.G1R6T | 0.23 | 14.22 | 61.826087 | Unknown protein |
| SSCG297 | Araip.6TY8W | 0.08 | 13.76 | 172 | PREDICTED: E3 ubiquitin-protein ligase BOI-like [*Glycine* *max*] |
| SSCG298 | Araip.B5WS3 | 0.26 | 13.48 | 51.8461538 | Non-specific lipid-transfer protein, putative |
| SSCG299 | Araip.R35MS | 0 | 13.4 | - | Unknown protein |
| SSCG300 | Araip.AVW8F | 0 | 13.14 | - | Unknown protein |
| SSCG301 | Aradu.JCT7W | 0.19 | 13.12 | 69.0526316 | Nodulin MtN21 /EamA-like transporter family protein |
| SSCG302 | Araip.CNB2V | 0.12 | 12.88 | 107.333333 | ATP-binding ABC transporter |
| SSCG303 | Aradu.31E79 | 0.03 | 12.87 | 429 | purine permease 4 |
| SSCG304 | Aradu.QT618 | 0.15 | 12.67 | 84.4666667 | glutamate-cysteine ligase |
| SSCG305 | Araip.W7U7R | 0.12 | 12.65 | 105.416667 | GDSL-like Lipase/Acylhydrolase superfamily protein |
| SSCG306 | Aradu.03P72 | 0 | 12.63 | - | PREDICTED: uncharacterized protein LOC102667487 isoform X2 [*Glycine* *max*] |
| SSCG307 | Araip.8D2WZ | 0 | 12.47 | - | PREDICTED: B3 domain-containing transcription factor FUS3-like [*Glycine* *max*] |
| SSCG308 | Aradu.N3N4U | 0 | 12.43 | - | Unknown protein |
| SSCG309 | Araip.FFN8X | 0.02 | 12.4 | 620 | purine permease 4 |
| SSCG310 | Aradu.2E3F6 | 0.14 | 12.39 | 88.5 | Protein kinase superfamily protein |
| SSCG311 | Aradu.PA53P | 0 | 12.16 | - | Stigma-specific Stig1 family protein |
| SSCG312 | Aradu.H8K0T | 0.1 | 12.09 | 120.9 | plant-specific B3-DNA-binding domain protein |
| SSCG313 | Aradu.C7AZ0 | 0 | 12.03 | - | Protein phosphatase 2C family protein |
| SSCG314 | Araip.2E2K8 | 0 | 12.03 | - | Sugar transporter SWEET |
| SSCG315 | Araip.NI8G1 | 0.04 | 11.96 | 299 | PREDICTED: putative pentatricopeptide repeat-containing protein At5g37570-like [*Glycine* *max*] |
| SSCG316 | Aradu.D5AUT | 0 | 11.9 | - | PREDICTED: low-temperature-induced 65 kDa protein-like [*Glycine* *max*] |
| SSCG317 | Araip.CCW4K | 0 | 11.85 | - | late embryogenesis abundant protein |
| SSCG318 | Aradu.RCQ63 | 0.03 | 11.76 | 392 | Cellulase (glycosyl hydrolase family 5) protein |
| SSCG319 | Araip.7JC2C | 0.11 | 11.75 | 106.818182 | YABBY transcription factor |
| SSCG320 | Araip.W7WHZ | 0.13 | 11.74 | 90.3076923 | PREDICTED: AP2-like ethylene-responsive transcription factor At1g16060-like [*Glycine* *max*] |
| SSCG321 | Araip.UJ49C | 0 | 11.47 | - | Unknown protein |
| SSCG322 | Araip.16PHC | 0 | 11.25 | - | plant invertase/pectin methylesterase inhibitor |
| SSCG323 | Araip.44NLV | 0 | 11.19 | - | Unknown protein |
| SSCG324 | Araip.VE0EE | 0.21 | 11.12 | 52.952381 | PREDICTED: protodermal factor 1-like isoform 1 [*Glycine* *max*] |
| SSCG325 | Araip.K8KF4 | 0 | 11.04 | - | PREDICTED: ethylene-responsive transcription factor 4-like [*Glycine max*] |
| SSCG326 | Aradu.XEX7M | 0.2 | 11 | 55 | basic helix-loop-helix (bHLH) DNA-binding superfamily protein |
| SSCG327 | Araip.YK7C2 | 0.08 | 10.91 | 136.375 | growth-regulating factor 5 |
| SSCG328 | Aradu.3BK8I | 0.08 | 10.8 | 135 | PREDICTED: zinc finger protein CONSTANS-LIKE 4-like [*Glycine* *max*] |
| SSCG329 | Araip.V2GHP | 0 | 10.77 | - | Unknown protein |
| SSCG330 | Aradu.Y4YT8 | 0 | 10.76 | - | Unknown protein |
| SSCG331 | Aradu.ZT2KF | 0.11 | 10.41 | 94.6363636 | zinc finger protein CONSTANS-LIKE 12-like [*Glycine* *max*] |
| SSCG332 | Araip.G10X7 | 0 | 10.31 | - | PREDICTED: uncharacterized protein LOC102667487 isoform X2 [*Glycine* *max*] |
| SSCG333 | Araip.K7W5L | 0 | 10.29 | - | GDSL-like Lipase/Acylhydrolase superfamily protein |
| SSCG334 | Araip.IY6V0 | 0.1 | 10.27 | 102.7 | receptor kinase 3 |
| SSCG335 | Araip.JB9A2 | 0 | 10.04 | - | PREDICTED: gibberellin 20 oxidase 2-like [*Glycine* *max*] |
| SSCG336 | Aradu.PW4PJ | 0.03 | 10.04 | 334.666667 | PREDICTED: adiponectin receptor protein 2-like isoform X3 [*Glycine* *max*] |
| SSCG337 | Aradu.T6Y1P | 0.05 | 9.97 | 199.4 | B3 DNA-binding domain protein |
