# Supplementary material for: Genome-wide mining seed-specific candidate genes from peanut for promoter cloning
Source: PLoS One. 2019 Mar 28;14(3):e0214025. doi: 10.1371/journal.pone.0214025 (PMC6438489; doi:10.1371/journal.pone.0214025)
Supplement: S3 Table — (DOCX) [file pone.0214025.s005.docx]

**S3 Table. Primers used in this study**

| **Genes** | **primers** | | **Functions** |
| --- | --- | --- | --- |
|  | **Forward** | **Reverse** |  |
| SSCG1/SSCG3 | ACATCTACAACCCTCAAGCTG | TGCCTTGCCTGCTCCCTTGG | RT-PCR |
| SSCG2/SSCG9 | ACATCTACAACCCTCAAGCTG | TGCCTTGCCTGCTCCCTTGG | RT-PCR |
| SSCG5/SSCG7 | ACATCTACAACCCTCAAGCTG | TGCCTTGCCTGCTCCCTTGG | RT-PCR |
| SSCG4/SSCG6 | ACATCTACAACCCTCAAGCTG | TGCCTTGCCTGCTCCCTTGG | RT-PCR |
| SSCG8 | CTTGTTGAGGACATTGC | GTGGATCCTTCCTTCCCCTC | RT-PCR |
| SSCG10/SSCG11 | TACATCCTCCTTTTGACCCC | CTCCACCATACACTATCCTC | RT-PCR |
| SSCG12/SSCG21 | CAGAGGGGTTTCCAGCAATA | GAAAAGAAGGGATGACGA | RT-PCR |
| SSCG13/SSCG44 | TCAATGAACTCAACCTCGTC | CGTAACACACTATGTGAAGA | RT-PCR |
| SSCG14 | GTTGAACCATTCGTGATAGC | GGTAATCCTCAATCACAAGG | RT-PCR |
| SSCG15/SSCG20 | GCCACGAAGACAAGGACG | TGAGAGTGCTCTTGTAAAGG | RT-PCR |
| SSCG16/SSCG27 | CGTGCGATTGGCTTTA | CAGAACATACAGAACCCCCC | RT-PCR |
| SSCG17 | GGCAGATACAGCAATGAAAG | AGCAGCAACTGTCTTGGGGC | RT-PCR |
| SSCG18/SSCG22 | CTTAGGTGGCGGTCCATACG | GGATGGGGAGAATGGTCCTTG | RT-PCR |
| SSCG19/SSCG24 | GCTGCTAATGTTGGTGCTTC | AATGAGTGGCAGTTCCGTAG | RT-PCR |
| SSCG23/SSCG37 | AAAGGAAGGTGTTGAAAGC | TTCATAGTGTCACGGTAGTG | RT-PCR |
| SSCG25 | ATGGGTCAATGGATAGCACT | TGCGGATAGAATCAGGAATG | RT-PCR |
| SSCG26/SSCG72 | GAATGAAGGAGGAATGGTGT | TGGTATCCTTCTTGATAGGTGG | RT-PCR |
| SSCG28/SSCG34 | TTTGCGGCGGTTTCTGCT | TCCAAGCCCTGATGTCAC | RT-PCR |
| SSCG29/SSCG30 | GGTGCTTACTCAAGGGTCAA | TCTCTCCTCCTTCTCTGCTT | RT-PCR |
| SSCG31/SSCG42 | TGGAGGGAGGTCTTGATGTT | GGGATGACTACAACTTGACC | RT-PCR |
| SSCG32 | TCCTTTTAGGGGTGGTTCTC | CTCCATAACCATCTCCTCCA | RT-PCR |
| SSCG33/SSCG50 | CAACAGCATCCTACCACCAC | TCAACAAGAGGCAGACCA | RT-PCR |
| SSCG35/SSCG59 | TGGAAGTGAGATGACAAGTG | TCAAGAGCCTAAATGCCC | RT-PCR |
| SSCG36/SSCG45 | ATCGCTGTTCCCCACG | TTGCCTTGCTTGCTCA | RT-PCR |
| SSCG38/SSCG46 | TGGTGGTTCTCCTGATTC | ATGAACGCTGAGGTTCG | RT-PCR |
| SSCG39/SSCG74 | TTACAAACCCAACCTGCTG | TGATGCGAGTTGGTTAGGGA | RT-PCR |
| SSCG40/SSCG57 | TGACATTCAATCCCAC | CTCCCTGTTGTTGCTG | RT-PCR |
| SSCG41/SSCG75 | CTGGATTCTTGACTGCTCTC | ACCTTTCTTCCATTTTCTCC | RT-PCR |
| SSCG43/SSCG94 | CTTCTTCCTCGTTCTCTTTC | TCCTCTGTATGTATCTGCCA | RT-PCR |
| SSCG47/SSCG71 | TGTTCTTGCCACCGTA | CGATTGTTATGCCTATTTCC | RT-PCR |
| SSCG48/SSCG91 | ACTTTCAACCGCTACAGAGA | TGTAGTCCCCTGAAGTTTCT | RT-PCR |
| SSCG49/SSCG62 | ATGGAGAAATCTGGCG | TGGCTGCTTCCGTTGT | RT-PCR |
| SSCG51/SSCG88 | ACGACGAAGAACGCAG | CATCACCCAACACACC | RT-PCR |
| SSCG52/SSCG58 | CTACCACGGCAAAACA | GCCCTCCAACTATCAG | RT-PCR |
| SSCG53/SSCG54 | ATGGGTTTATTCGGGGTG | CAACCCCCCAATGGATTC | RT-PCR |
| SSCG56/SSCG90 | GCAGTATGTTGAGCCGAC | ACCCAGCATTGAAAGCCA | RT-PCR |
| SSCG60 | CTCAAACCCGTGAAACAG | GAAAAGGTGCCCTGAGTA | RT-PCR |
| SSCG61 | ACTGAGAGGAGTGCCAAA | CTCACCACTCTCCAAACA | RT-PCR |
| SSCG63/SSCG103 | TGCCTCAACAAGTTCATC | TAGTCGGAGTCACGGTAG | RT-PCR |
| SSCG64 | ACTTACGCCACTGCTGAA | ACTGACTCCCACTGGAAA | RT-PCR |
| SSCG65 | TCTGCTACTTGTGTGCCT | GGTTTGCCCTTAGAGTTC | RT-PCR |
| SSCG66/SSCG81 | TGCTGTGAAAGGATTGGC | CCCTTCAACATTCTTGTG | RT-PCR |
| SSCG67/53/54 | CGCTGCCAACATTTTACC | GCAAACCACATAACCGCA | RT-PCR |
| SSCG68/SSCG85 | ATACTCTAACAGGGAAAGGC | TGAGAACAAATGATGAGCCAC | RT-PCR |
| SSCG69/SSCG92 | AAAGGACAAGACAGCCCA | TGCCTTTAACCTTTTCCC | RT-PCR |
| SSCG70 | CTGATGAATCGTCCAAGG | CCCTCTTTTTCTCGTTCT | RT-PCR |
| SSCG72 | GCAGGGATGGTTGAGAAT | TGCGTCTCTTTGGTATCC | RT-PCR |
| SSCG73/SSCG107 | GCAACATTCACAGCCTCT | GGCAAAATAGCGAGTGTT | RT-PCR |
| SSCG76 | TGCTTGTGATGTTGCTCT | CACTTGGGTTTGTTGCCA | RT-PCR |
| SSCG77 | GGCGAAAGAGTATGGTGA | ACCCACTCCATTTGCTCA | RT-PCR |
| SSCG78 | GCCTGACAAACCACACAA | CTTTAGGCGATGGGTAGA | RT-PCR |
| SSCG79 | GAAGGAAGGGAAGGACAC | GTCAGATGCTTTCAAGGC | RT-PCR |
| SSCG80 | CGTGTCATTACGGAAAGC | ATGTCCGCCTTGTTTCTA | RT-PCR |
| SSCG82 | TGGCAGTCCTTCAATACC | GTGGGTTTCTTCAAGTGG | RT-PCR |
| SSCG83 | TGTGCTTGTGATTGCTTC | CGTGTTTAGCAGTTGGGT | RT-PCR |
| SSCG84 | TTTGTATCTTTCGGGTCG | TCAACAAGGAACTCCAAG | RT-PCR |
| SSCG86 | GTCTACCTTGGCTTCTCT | GTTGGTGGGTTGCTTGAA | RT-PCR |
| SSCG87/SSCG100 | TTGGGCTTTCAGCACTAC | GGATGGAGAAGCCTTTTG | RT-PCR |
| SSCG89/SSCG93 | TATGGTGTTGGCGAAGGA | TTGCTCTGCCAGTTCTGA | RT-PCR |
| SSCG95 | TGGAGAAGGATTCCGATG | CCATCACTGCTCCCTTCT | RT-PCR |
| SSCG96 | TCTTATCACCATCACCGC | AGGAGAAGGGGAACTTGA | RT-PCR |
| SSCG97 | CAAATCTGAACAACGGTG | TCAATCTCACAACGACCT | RT-PCR |
| SSCG98 | GAGAAGTTTGACAAGCGT | TCTTGCTTCTCCTTGCCT | RT-PCR |
| SSCG99 | GGAAATCTGGCTCAAAAG | GCCCACTTCACCAATACC | RT-PCR |
| SSCG101 | CCGACTTTGCTTCTTCCA | ACAGCAACCTAACCACGC | RT-PCR |
| SSCG102 | CGAAGAACAGCAAGAGTA | CAGATGGAGGAGGTTGAT | RT-PCR |
| SSCG104/SSCG108 | GGATTTTGGGCAAGAGAC | ATCCACCTACCACCTTGC | RT-PCR |
| SSCG105 | AAGGGCATCTTGGAACAC | GGTCTGCGGCTGTTTTCT | RT-PCR |
| SSCG106 | GGCATCTCATGACCAAAG | ATTGTCCCCAGTAGTCCT | RT-PCR |
| GUS | GTGACAAAAACCACCCAAG | CTTTCTTGTTACCGCCAAC | Transgenic detection |
| AHSSP29 | AAGCTTGTATCATAGATTCAGTCGTCGTCG | GGATCCCACAAGAAAAGGAGGAGGTGAAAGGAATAC | Amplification for AHSSP29 promoter |

Underlined is *Hind*III and *Bam*HI restriction enzyme site.
